# Vaccinia Virus–Encoded Ribonucleotide Reductase Subunits Are Differentially Required for Replication and Pathogenesis

**DOI:** 10.1371/journal.ppat.1000984

**Published:** 2010-07-08

**Authors:** Don B. Gammon, Branawan Gowrishankar, Sophie Duraffour, Graciela Andrei, Chris Upton, David H. Evans

**Affiliations:** 1 Department of Medical Microbiology and Immunology, University of Alberta, Edmonton, Alberta, Canada; 2 Laboratory of Virology and Rega Institute for Medical Research, Katholieke Universiteit Leuven, Leuven, Belgium; 3 Biochemistry and Microbiology, University of Victoria, Victoria, British Columbia, Canada; University of Florida, United States of America

## Abstract

Ribonucleotide reductases (RRs) are evolutionarily-conserved enzymes that catalyze the rate-limiting step during dNTP synthesis in mammals. RR consists of both large (R1) and small (R2) subunits, which are both required for catalysis by the R1_2_R2_2_ heterotetrameric complex. Poxviruses also encode RR proteins, but while the Orthopoxviruses infecting humans [e.g. vaccinia (VACV), variola, cowpox, and monkeypox viruses] encode both R1 and R2 subunits, the vast majority of Chordopoxviruses encode only R2 subunits. Using plaque morphology, growth curve, and mouse model studies, we investigated the requirement of VACV R1 (I4) and R2 (F4) subunits for replication and pathogenesis using a panel of mutant viruses in which one or more viral RR genes had been inactivated. Surprisingly, VACV F4, but not I4, was required for efficient replication in culture and virulence in mice. The growth defects of VACV strains lacking F4 could be complemented by genes encoding other *Chordopoxvirus* R2 subunits, suggesting conservation of function between poxvirus R2 proteins. Expression of F4 proteins encoding a point mutation predicted to inactivate RR activity but still allow for interaction with R1 subunits, caused a dominant negative phenotype in growth experiments in the presence or absence of I4. Co-immunoprecipitation studies showed that F4 (as well as other *Chordopoxvirus* R2 subunits) form hybrid complexes with cellular R1 subunits. Mutant F4 proteins that are unable to interact with host R1 subunits failed to rescue the replication defect of strains lacking F4, suggesting that F4-host R1 complex formation is critical for VACV replication. Our results suggest that poxvirus R2 subunits form functional complexes with host R1 subunits to provide sufficient dNTPs for viral replication. Our results also suggest that R2-deficient poxviruses may be selective oncolytic agents and our bioinformatic analyses provide insights into how poxvirus nucleotide metabolism proteins may have influenced the base composition of these pathogens.

## Author Summary

Efficient genome replication is central to the virulence of all DNA viruses, including poxviruses. To ensure replication efficiency, many of the more virulent poxviruses encode their own nucleotide metabolism machinery, including ribonucleotide reductase (RR) enzymes, which act to provide ample DNA precursors for replication. RR enzymes require both large (R1) and small (R2) subunit proteins for activity. Curiously, some poxviruses only encode R2 subunits. Other poxviruses, such as the smallpox vaccine strain, vaccinia virus (VACV), encode both R1 and R2 subunits. We report here that the R2, but not the R1, subunit of VACV RR is required for efficient replication and virulence. We also provide evidence that several poxvirus R2 proteins form novel complexes with host R1 subunits and this interaction is required for efficient VACV replication in primate cells. Our study explains why some poxviruses only encode R2 subunits and identifies a role for these proteins in poxvirus pathogenesis. Furthermore, we provide evidence that mutant poxviruses unable to generate R2 proteins may become entirely dependent upon host RR activity. This may restrict their replication to cells that over-express RR proteins such as cancer cells, making them potential therapeutics for human malignancies.

## Introduction

Critical for the replication of all organisms and DNA viruses is the conversion of ribonucleotides to deoxynucleotides to serve as building blocks for genome synthesis and repair. Ribonucleotide reductase (RR) is a key enzyme involved in this process, catalyzing the reduction of rNDPs to dNDPs [1], [2]. RRs can be grouped into one of three classes, based on their requirement for oxygen and the mechanism by which a catalytically-important thiyl radical is generated [1]. Mammals typically encode class I RR proteins while class II and III proteins are found only in microorganisms [1], [3]. Class I RR enzymes are assembled from both large (R1; 80–100 kDa) and small (R2; 37–44 kDa) protein subunits, which associate to form enzymatically-active R1_2_R2_2_ tetrameric complexes [1]. These complexes require oxygen to generate a tyrosyl radical found within R2 subunits [1], [4], which is ultimately transferred to R1 subunits to generate a thiyl radical used in rNDP reduction. Transfer of the tyrosyl radical from R2 to R1 subunits is thought to occur through a “radical transfer pathway” that uses a series of at least eleven highly-conserved amino acid residues to promote long-range electron transfer [4], [5], [6], [7], [8]. Mutant proteins containing amino acid substitutions at either the tyrosine involved in radical formation [9] or any of the proposed transfer pathway residues [4], [6], [8], [10], [11] form inactive RR complexes, indicating that both radical formation and transfer are required for catalysis.

**Figure 1:**
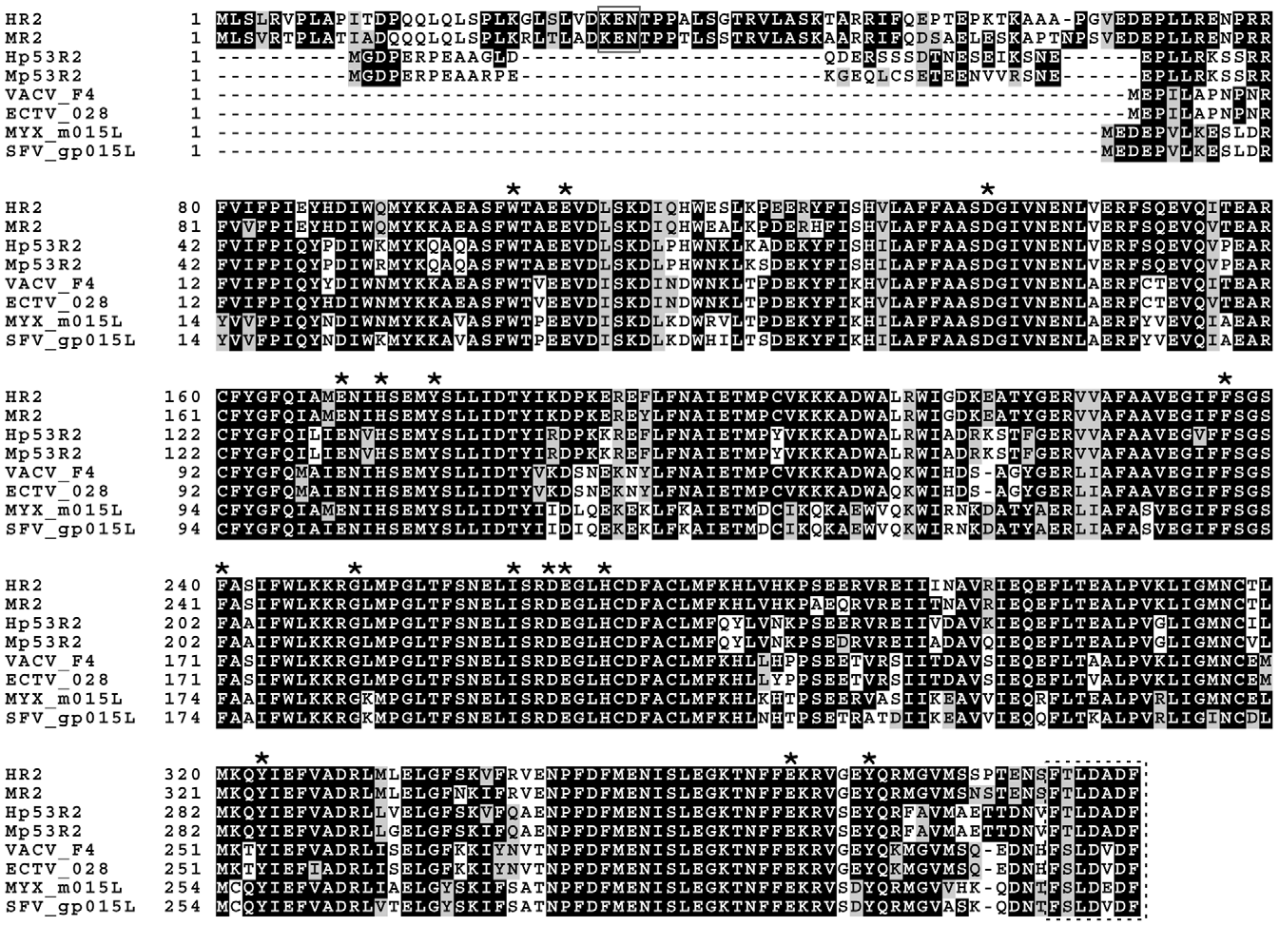
Alignment of cellular and poxvirus small RR subunits. Alignment of human R2 (HR2; Genbank accession: NP_001025.1), mouse R2 (MR2; Genbank accession: NP_033130.1), human p53R2 (Hp53R2; Genbank accession: BAD12267.1), mouse p53R2 (Mp53R2; Genbank accession: Q6PEE3.1) as well as R2 subunits encoded by vaccinia (VACV; Genbank accession: AAO89322.1), ectromelia (ECTV; Genbank accession: NP_671546.1), myxoma (MYXV; Genbank accession: NP_051729.1), and Shope fibroma (SFV; Genbank accession: NP_051904.1) viruses was performed using ClustalW. Asterisks indicate catalytically-important residues [61]. The solid box indicates the “KEN” box found in cellular R2 proteins [15]. The dashed box indicates the putative R1-binding domain.

Mammalian cells encode a single R1 gene that is only transcribed during S-phase [12]. However, due to the long half-life (∼15 h) of R1 proteins, R1 levels remain essentially constant throughout the cell cycle [13]. The primary small subunit, R2, is also only expressed during S-phase [12], [14] however, this protein has a short half-life (∼3 h) and is rate-limiting for R1-R2 complex formation [13]. The short half-life of R2 is due to its polyubiquitination by the anaphase-promoting complex (APC)-Cdh1 ubiquitin ligase, which leads to its degradation during mitosis [15]. This degradation is dependent upon APC-Cdh1 recognition of a “KEN” box sequence in the N-terminus of R2 (Figure 1). Mammals also encode a second small subunit, p53R2, so named because its elevated expression in response to DNA damage is dependent upon the tumor suppressor p53 [16]. Although p53R2 is 80–90% identical to cellular R2 and can form active complexes with R1 [17], it lacks ∼33 N-terminal amino acid residues found in R2, including those containing the KEN box (Figure 1) [15]. The absence of the KEN box sequence likely explains why p53R2 levels are relatively constant throughout the cell cycle in the absence of DNA damage [18]. It has been hypothesized that p53R2 plays some role in supporting mitochondrial DNA synthesis and/or DNA repair outside of S-phase [17], [18], [19], [20]. Therefore, despite their similarity, R2 and p53R2 appear to be differentially regulated and probably serve different purposes during the cell cycle.

Many Chordopox-, herpes-, asfra- and iridoviruses, also encode their own class I RR proteins [21], [22], [23], [24], [25]. These enzymes are generally thought to support viral replication since ribonucleotide reduction is normally the rate-limiting step in *de novo* dNTP biogenesis [26]. Although most Chordopoxviruses encode RR proteins, many only encode one of the two RR subunits with a clear bias towards the conservation of R2 proteins (Table S1). Only the Suipox- and Orthopoxviruses contain both R1 and R2 genes. The latter group contains viruses of medical importance including variola virus, the causative agent of smallpox, as well as monkeypox and cowpox viruses, which are responsible for zoonoses in humans [27], [28]. Most understanding of poxvirus RR proteins comes from studies with another *Orthopoxvirus*, vaccinia virus (VACV).

Slabaugh and Mathews [29] were the first to show that RR activity increased in VACV-infected cells and subsequent studies identified the *I4L* [25], [30] and *F4L* [24] genes as those encoding the 87 kDa I4 (R1) and 37 kDa F4 (R2) proteins, respectively. Biochemical studies showed that VACV and cellular RR enzymes share many features, including a similar tertiary architecture, similar pH dependence, allosteric modulation of activity by nucleotides, and comparable specific activities on most rNDP substrates [31], [32], [33]. However, unlike cellular RR, the viral enzyme is less sensitive to allosteric modulation and shows little activity on UDP substrates, indicating that viral and cellular RR enzymes also differ in important ways [33]. The similarities between VACV and mammalian RR are not unexpected given that VACV (and other poxvirus) RR subunits typically share >70% sequence identity with their mouse and human homologs (Figure 1).

Previous studies have shown that inactivating VACV *I4L* does not affect plaque size and that *I4L*-deficient mutant strains replicate their DNA and produce viral particles to levels comparable to wild-type VACV in cell culture [34], [35]. Due to these observations, the *I4L* locus has been suggested to be an excellent site for insertion of foreign genes into VACV [36]. Furthermore, *I4L* mutants are only mildly attenuated in their virulence in mouse models, exhibiting an ∼10-fold increase in lethal dose 50 (LD_50_) values when compared to wild-type virus [34]. Paradoxically, another group reported that targeted inactivation of *F4L* attenuated VACV in mice by ∼1000-fold compared to wild-type virus [37], but the reason for this attenuation was unknown. Although these were separate studies using different strains of VACV, they suggested that the F4 subunit is more important for virus replication and pathogenesis than I4, despite the fact that both subunits are needed for RR activity [32].

We initiated our studies of VACV RR because VACV recombination appears to be catalyzed by poxviral DNA polymerases *in vivo* [38], and we wanted to determine if perturbing dNTP pools would affect this process. However, it soon became apparent that some of the mutant strains we generated exhibited previously uncharacterized replication defects. This prompted us to revisit how VACV RR affects viral replication and pathogenesis. To do this, we generated a panel of mutant strains containing mutations in the VACV RR genes. We also generated strains lacking a functional *J2R* (thymidine kinase or TK) gene, thus unable to access the parallel viral salvage pathway of dTTP biogenesis [39]. Our studies show that both the VACV R1 and R2 proteins can form a diversity of virus-virus and virus-host protein-protein interactions *in vivo*, but that the VACV R2 subunit is far more critical for VACV replication and pathogenicity than is the R1 subunit. Our model suggests that poxvirus R2 subunits form active complexes with host R1 proteins in order to ensure a sufficient dNTP supply to support viral replication. This model is substantiated by previous biochemical studies that found a chimeric RR enzyme consisting of VACV F4 and mouse R1 (MR1) to be more active than strictly viral or mouse RR complexes [33]. Our studies also provide insights into why poxviruses have often conserved their R2 but not their R1 genes (Table S1). To our knowledge this is the first report of a chimeric, virus-host RR forming *in vivo*. Our study provides further evidence that poxviruses recruit cellular enzymes, in addition to those previously identified such as topoisomerase II [40] and DNA ligase I [41], to support viral replication. Our bioinformatic analysis of other large DNA viruses suggests that recruitment of host RR subunits may represent a more widespread viral strategy to parasitize host nucleotide biosynthetic machinery.

## Results

### Generation of VACV RR mutant strains

**Figure 2:**
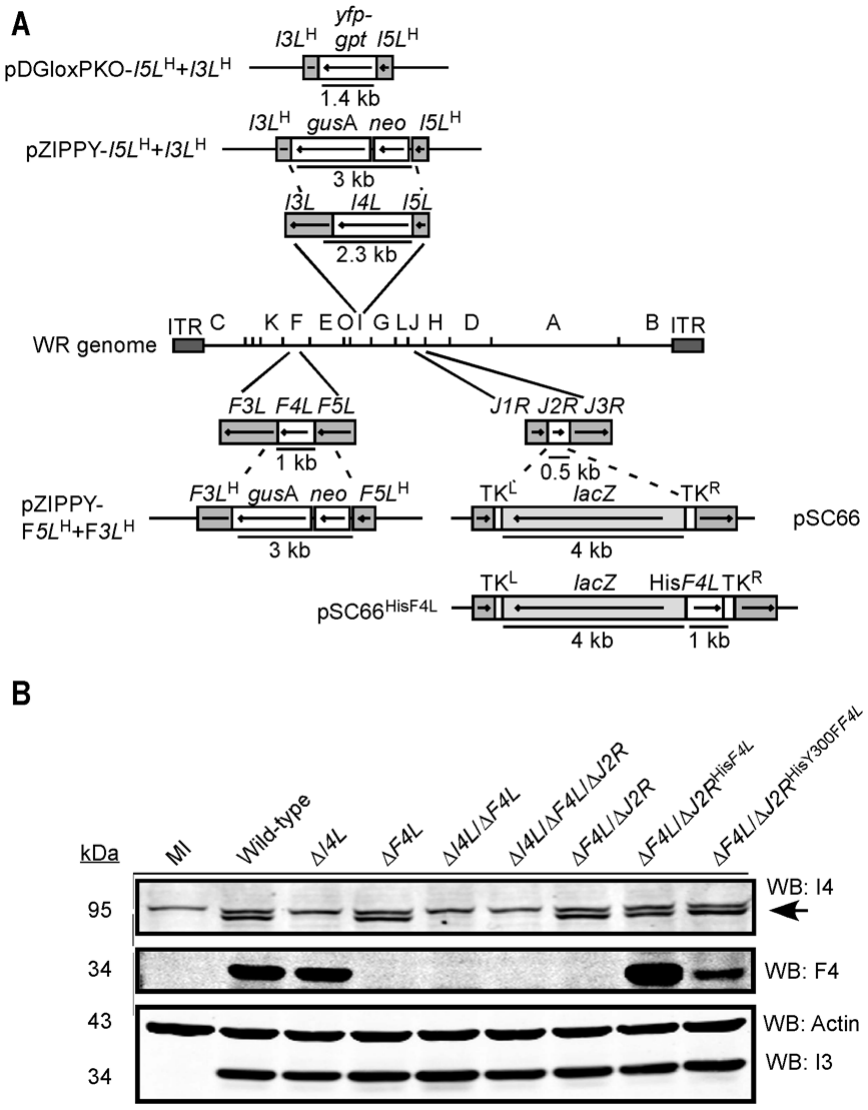
Construction of recombinant VACV strains. (A) Schematic of strategy used to inactivate *I4L*, *F4L* and/or *J2R* function in VACV using homologous recombination. The Δ*I4L* and Δ*F4L* strains were generated using the pZIPPY-NEO/GUS vector [109]. The Δ*I4L*/Δ*F4L* strains were generated with the pDGloxPKO^INV^ vector which replaces *I4L* sequence with a *yfp*-*gpt* fusion cassette flanked by loxP sites (not shown). A pSC66 shuttle vector was used to insert several different ectopic genes into the *J2R* locus although only pSC66^His*F4L*^ is shown as an example. See Materials and Methods and Text S1 for further details. (B) Western blot analysis of I4 and F4 expression. Proteins were extracted from HeLa cells 8 h post-infection with the indicated strains, size fractioned and blotted using the indicated antibodies. Blotting for the constitutively-expressed viral I3 protein and cellular actin served as loading controls. Note that in I4 blots the lower band (indicated by an arrow) represents I4 (87.6 kDa) and the upper band is due to cross-reactivity with HR1 (89.9 kDa).

A series of mutant strains were generated in which one (Δ*I4L*; Δ*F4L*) or both (Δ*I4L*/Δ*F4L*) of the VACV RR genes were deleted from the viral genome (Figure 2A). We also constructed VACV encoding an insertional inactivation of the *J2R* (TK) gene in combination with Δ*I4L* and/or Δ*F4L* mutations generating Δ*I4L*/Δ*F4L*/*ΔJ2R* and Δ*F4L*/*ΔJ2R* strains. These strains provide insights into the relative biological importance of the *de novo* (RR-dependent) and salvage (TK-dependent) pathways in VACV replication. We also constructed VACV Δ*F4L* strains encoding a His_6_-tagged *F4L* gene, or a His_6_-tagged *F4L* gene encoding a Y300F amino acid substitution, inserted into the *J2R* locus. These viruses are referred to as VACV strains Δ*F4L*/Δ*J2R* ^His*F4L*^ and Δ*F4L*/Δ*J2R* ^HisY300F*F4L*^, respectively. The marker rescue strategies used to generate these mutant strains are depicted in Figure 2A.

PCR-based analysis confirmed the deletion or inactivation of the targeted loci in constructed VACV strains (data not shown). Western blotting confirmed the presence or absence of viral RR subunit expression in each of the isolates (Figure 2B). The Δ*F4L*/Δ*J2R* ^His*F4L*^ strain appeared to express elevated levels of F4 compared to wild-type virus, whereas the Δ*F4L*/Δ*J2R* ^HisY300F*F4L*^ strain had slightly reduced F4 expression (Figure 2B). The former case is likely a result of the *F4L* gene being under the control of an early/late promoter present on the pSC66 transfer vector whereas the endogenous *F4L* promoter is activated only at early times during infection [42]. The lower F4 expression of the Δ*F4L*/Δ*J2R* ^HisY300F*F4L*^ strain is likely due to its poor replication in culture (see below). These and other VACV strains are summarized in Table 1. See Text S1 for details of virus construction.

**Table 1:** Major VACV strains used in this study.

Strain^1^	*I4L* locus^2^	*F4L* locus^2^	*J2R* locus^2^
Wild-type (WR)	+	+	+
Δ*I4L*	−(*neo*; *gusA*)	+	+
Δ*F4L*	+	−(*neo*; *gusA*)	+
Δ*J2R*	+	+	−(*lacZ*)
Δ*I4L*/Δ*F4L*	−(*yfp-gpt*)	−(*neo*; *gusA*)	+
Δ*I4L*/Δ*F4L*/Δ*J2R*	−(*yfp-gpt*)	−(*neo*; *gusA*)	−(*lacZ*)
Δ*F4L*/Δ*J2R*	+	−(*neo*; *gusA*)	−(*lacZ*)
Δ*F4L*/Δ*J2R* ^His*F4L*^	+	−(*neo*; *gusA*)	−(*lacZ*; His*F4L*)
Δ*F4L*/Δ*J2R* ^HisY300F*F4L*^	+	−(*neo*; *gusA*)	−(*lacZ*; HisY300F*F4L*)
Δ*I4L*/Δ*F4L*/Δ*J2R* ^His*F4L*^	−(*yfp-gpt*)	−(*neo*; *gusA*)	−(*lacZ*; His*F4L*)
Δ*I4L*/Δ*F4L*/Δ*J2R* ^HisY300F*F4L*^	−(*yfp-gpt*)	−(*neo*; *gusA*)	−(*lacZ*; HisY300F*F4L*)
Δ*F4L*/Δ*J2R* ^His*F4L*ΔR1BD^	+	−(*neo*; *gusA*)	−(*lacZ*; His*F4L*ΔR1BD)
Δ*F4L*/Δ*J2R* ^HisY300F*F4L*ΔR1BD^	+	−(*neo*; *gusA*)	−(*lacZ*; HisY300F*F4L*ΔR1BD)
Δ*F4L*/Δ*J2R* ^HisECTVR2^	+	−(*neo*; *gusA*)	−(*lacZ*; HisECTVR2)
Δ*F4L*/Δ*J2R* ^HisMYXR2^	+	−(*neo*; *gusA*)	−(*lacZ*; HisMYXR2)
Δ*F4L*/Δ*J2R* ^HisSFVR2^	+	−(*neo*; *gusA*)	−(*lacZ*; HisSFVR2)
Δ*I4L*/Δ*J2R* ^Flag*I4L*^	−(*neo*; *gusA*)	+	−(*lacZ*; HisSFVR2)
Δ*J2R* ^FlagHR1^	+	+	−(*lacZ*; FlagHR1)
Δ*J2R* ^HisHp53R2^	+	+	−(*lacZ*; HisHp53R2)
Δ*F4L*/Δ*J2R* ^HisHp53R2^	+	−(*neo*; *gusA*)	−(*lacZ*; HisHp53R2)

1 All strains were generated in the Western Reserve (WR) strain of VACV.

2 “+” indicates locus is intact and “−” indicates locus is disrupted. Marker genes and inserted viral or human genes present at disrupted loci are in parentheses. Abbreviations: His, His_6_ epitope tag; Flag, Flag epitope tag; R1BD, R1-binding domain; VACV, vaccinia virus; ECTV, ectromelia virus; MYX, myxoma virus; SFV, Shope fibroma virus; HR1, human R1; Hp53R2, human p53R2. See Materials and Methods and Text S1 for further details.

### Δ*F4L* strains exhibit a small plaque phenotype

**Figure 3:**
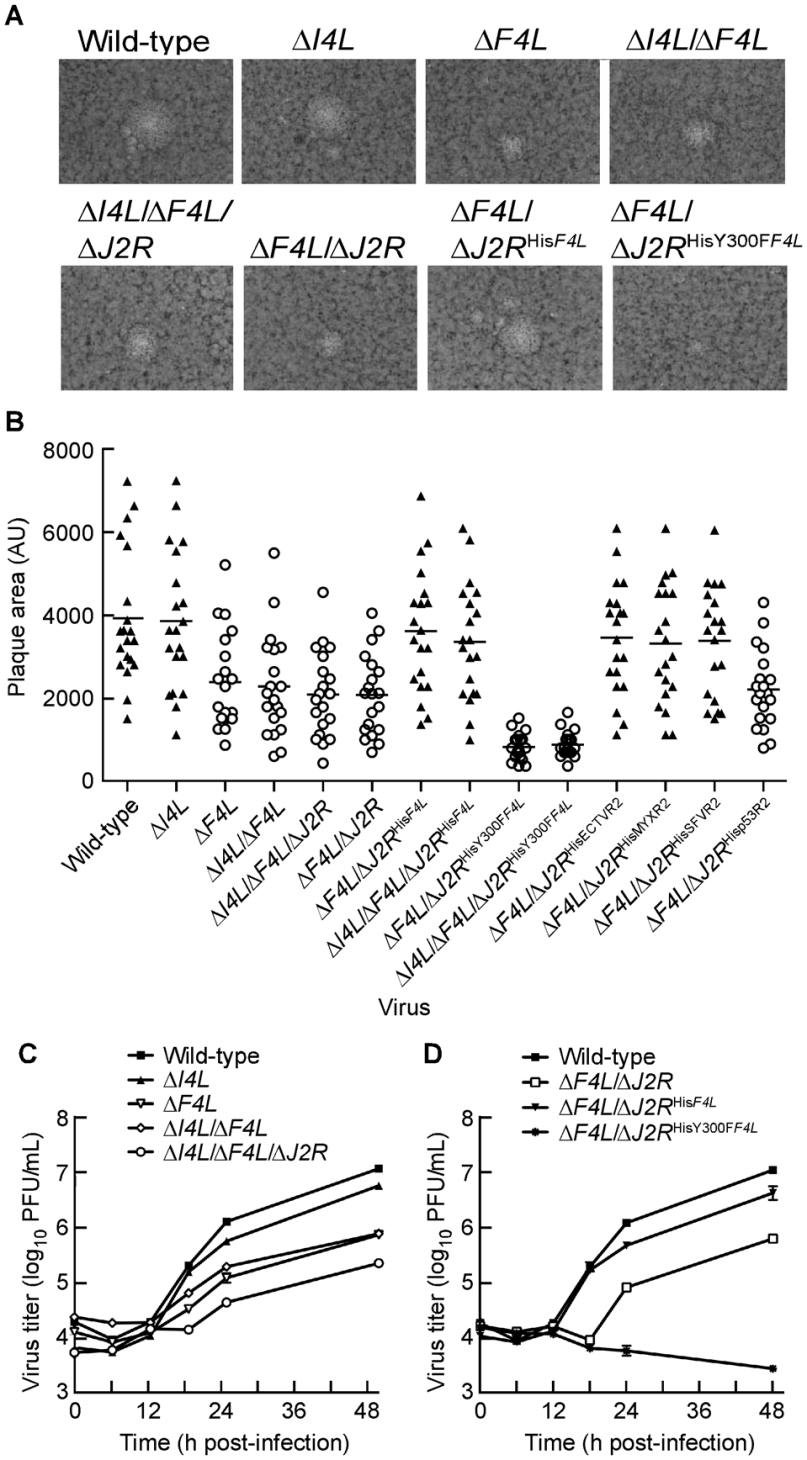
Δ*F4L* strains exhibit a small plaque phenotype and impaired replication *in vitro*. (A) Representative plaques formed by each of the indicated strains 48 h post-infection on BSC-40 cells. (B) Scatter plots illustrating independent (n = 20) as well as mean (horizontal bar) plaque area measurements in arbitrary units (AU) for each of the indicated strains. Open circles indicate that the mean plaque area was statistically different (P<0.05) from wild-type virus based on a one-way ANOVA. (C) and (D) virus growth in HeLa cells infected with each of the indicated strains at a MOI of 0.03. Viruses were harvested at the indicated time points and tittered on BSC-40 cells. Note that experiments presented in (C) and (D) were done in parallel but are presented in two graphs for clarity. Thus, the wild-type curve is identical in both graphs. Symbols represent mean titers from three independent experiments and error bars represent SE. Some bars are approximately the same size as the symbols.

Plaque size and morphologies of the generated strains were analyzed on BSC-40 cells as an initial step to characterize their growth properties. The wild-type and Δ*I4L* strains exhibited similar plaque morphologies. These plaques typically had large central clearings and were accompanied by smaller secondary plaques that are formed when extracellular enveloped virus are released from infected cells and initiate new infections near primary plaque sites (Figure 3A). Upon measurement of primary plaque areas, no significant differences were found between wild-type and the Δ*I4L* strain (Figure 3B). In contrast, the Δ*F4L*, Δ*F4L*/Δ*J2R*, and Δ*I4L*/Δ*F4L*/Δ*J2R* strains all produced significantly smaller plaques (P<0.05) that were only 55–60% of the plaque size exhibited by wild-type virus (Figure 3B). In addition, the primary plaques produced by Δ*F4L* strains were typically devoid of nearby secondary plaques (Figure 3A). Incorporation of a His_6_-tagged form of *F4L* into the TK locus appeared to complement Δ*F4L* strain replication as the Δ*F4L*/Δ*J2R* ^His*F4L*^ strain displayed plaques characteristic of wild-type virus in terms of size and the presence of secondary plaques (Figure 3A and B). Strikingly, Δ*F4L* strains rescued with a His_6_-tagged *F4L* gene encoding the Y300F substitution produced plaques that were not only significantly smaller than wild-type virus [(P<0.05); Figure 3B], but were only 35–40% the size of plaques produced by any of the strains with *F4L* deleted and these differences were statistically significant (P<0.05). These results suggested that deletion of *F4L* has a more detrimental effect on plaque size than deletion of *I4L*. They further suggested that re-introduction of a His_6_-tagged *F4L* gene into the TK locus can rescue the small plaque phenotype of Δ*F4L* strains. However, this rescue effect is lost and the Δ*F4L* strain replication defect is exacerbated when the re-introduced F4 protein encodes the Y300F amino acid substitution.

Y300 represents a highly-conserved tyrosine residue found in essentially all mammalian small RR subunits (Figure 1). The homologous residue in mouse R2 (MR2; Y370) is required for the transfer of radicals from MR2 to MR1 subunits, which is necessary for catalysis [4]. A Y370F substitution abolishes catalysis but does not impede physical interaction of MR1 and MR2 subunits [4]. The same substitution of the homologous tyrosine residue in human p53R2 (Hp53R2) also abolishes RR activity of Human R1 (HR1)-Hp53R2 complexes [43]. Therefore, the Y300F substitution in F4 is predicted to inhibit catalysis while still allow for R1-R2 subunit interaction. These predicted properties of the Y300F F4 protein may explain the dominant negative-like phenotype exhibited by the Δ*F4L*/Δ*J2R* ^HisY300F*F4L*^ strain.

### Δ*F4L* strains exhibit impaired replication kinetics in culture

We also examined the growth kinetics of these mutant strains in HeLa cells. As previously reported [34], deleting *I4L* had little effect on viral replication, with the Δ*I4L* strain replicating to titers that were 2-fold lower than those produced by wild-type virus 48 h post-infection (Figure 3C). In contrast, large (>5-fold) differences between wild-type and Δ*F4L* strains were readily apparent by 18 h post-infection. This trend continued to the end of the experiment, with the wild-type strain producing ∼15–50-fold more virus than Δ*F4L* strains 48 h post-infection. This growth defect could be complemented with a His_6_-tagged *F4L* gene but not if this gene encoded the Y300F substitution (Figure 3D). In fact, the Δ*F4L*/Δ*J2R* ^HisY300F*F4L*^ strain was unable to undergo productive replication in HeLa cells (Figure 3D). These results suggested that deletion of the *F4L* gene impairs VACV replication to a higher degree than deletion of *I4L*, and that concomitant deletion of *F4L* and *J2R* does not impede replication further (Figure 3C and 3D). Furthermore, the fact that one can rescue the Δ*F4L* growth defect with a His_6_-tagged form of *F4L* inserted at the TK locus, implies that the defect seen in a Δ*F4L* strain is not due to other possible idiosyncratic effects caused by deleting the *F4L* locus (Figure 3D). Finally, these studies further illustrate the dominant negative effect on virus growth imposed by a catalytically-inactive, Y300F-substituted F4 protein.

One explanation for the properties of virus encoding a Y300F-substituted F4 protein is that the mutant protein could be competing with cellular R2 proteins for binding to cellular and/or viral R1 subunits. However, the studies shown in Figure 3C and D suggested that deleting *I4L* does not result in significant replication defects and so the dominant negative phenotype was likely mediated by interaction with cellular R1 proteins. To rule out a role for I4 interaction in this dominant negative phenotype, the His_6_-tagged wild-type or Y300F-encoding *F4L* gene was inserted into the *J2R* locus of Δ*I4L*/Δ*F4L* strains. The Δ*I4L*/Δ*F4L*/Δ*J2R* ^His*F4L*^ strain produced plaques indistinguishable in size from those formed by wild-type virus (P>0.05; Figure 3B). However, deleting *I4L* had no further effects on the plating properties of the Δ*I4L*/Δ*F4L*/Δ*J2R* ^HisY300F*F4L*^ strain. This strain still produced plaques that were significantly smaller than those produced by wild-type (P<0.05) or Δ*F4L* strains (P<0.05) and were not significantly different from Δ*F4L*/Δ*J2R* ^HisY300F*F4L*^ virus plaques (P>0.05; Figure 3B). These observations implied that the plaque properties of Δ*F4L* strains are not influenced by the presence or absence of *I4L*.

We also tested the ability of other, His_6_-tagged *Chordopoxvirus* or host R2 proteins to rescue the small plaque phenotype of the Δ*F4L* strain. The R2 genes encoded by ECTV, MYXV and SFV R2 genes were all able to rescue the small plaque phenotype, but interestingly the Hp53R2 gene failed to rescue this phenotype (Figure 3B). These results implied that *Chordopoxvirus* R2 proteins have conserved a specific function and/or activity level that is not recapitulated by Hp53R2.

### VACV DNA synthesis is impaired in cells infected with a Δ*F4L* strain

**Figure 4:**
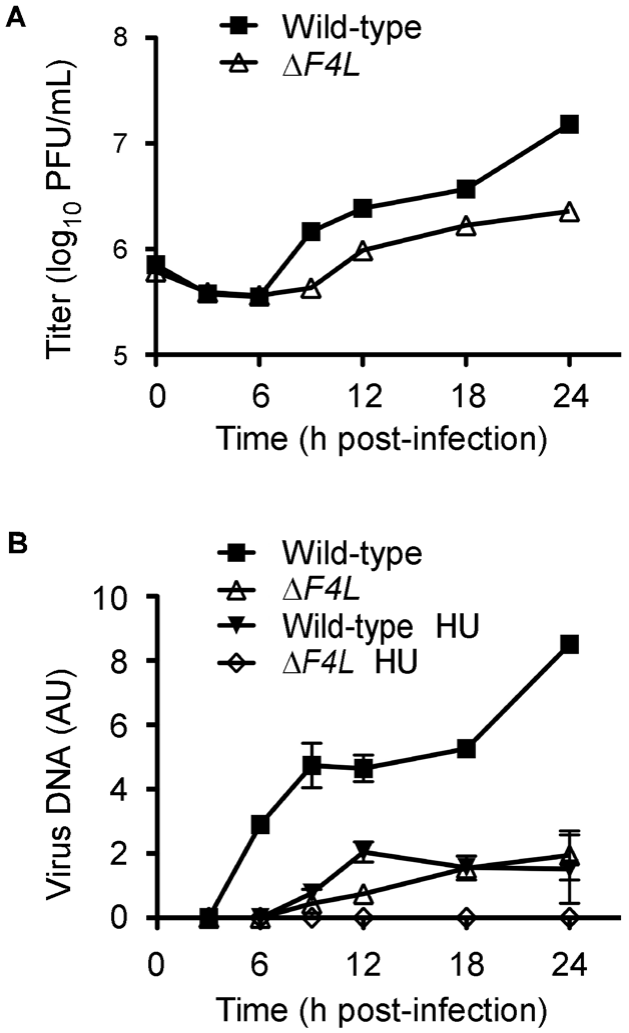
A VACV Δ*F4L* strain exhibits impaired growth and DNA replication kinetics in BSC-40 cells. (A) Growth curve analysis of wild-type and Δ*F4L* strains in BSC-40 cells infected at a MOI of 2. Symbols represent mean titers from three independent experiments and error bars represent SE, although some bars are approximately the same size as the symbols. (B) Viral DNA accumulation during infection with the indicated strains. Parallel samples from (A) were analyzed for viral DNA content expressed in arbitrary units (AU). Symbols represent mean DNA content from two independent experiments and error bars represent error of the mean. Some bars are approximately the same size as the symbols. “HU” indicates where 0.5 mM hydroxyurea was added to the culture media.

We hypothesized that the reduced replication of the Δ*F4L* strains was due to impaired genome replication. This is because RR plays a key role in dNTP biogenesis and our initial studies found that Δ*F4L* (Figure S1A), but not Δ*I4L* strains (Figure S1B), exhibited reduced late gene expression, which is a common consequence of defects in DNA replication. To test this hypothesis, BSC-40 cells were infected with wild-type or Δ*F4L* viruses and genome replication was measured in parallel with viral yields. The results of these experiments are shown in Figure 4. As in HeLa cells, the Δ*F4L* strain exhibited impaired replication kinetics in BSC-40 cells, generating only 15% of the total titer observed with the wild-type strain at 24 h post-infection (Figure 4A). This growth defect was associated with impaired DNA synthesis, with the Δ*F4L* strain exhibiting an ∼3 h delay in genome synthesis as well as an ∼5-fold reduction in DNA production at 24 h post-infection when compared to wild-type infections (Figure 4B). We also tested what effect the drug hydroxyurea (HU) would have on these strains, since previous studies have correlated HU resistance with changes in F4 expression [44]. Addition of 0.5 mM HU to Δ*F4L* strain-infected cultures completely blocked virus DNA synthesis. In contrast, wild-type virus still produced detectable amounts of genomic DNA, albeit with delayed kinetics, at levels comparable to what is seen in cells infected with the Δ*F4L* strain in the absence of HU (Figure 4B). These results suggested that the reduced yields observed with Δ*F4L* strains are at least partially due to impaired genome synthesis. Furthermore, because sensitivity to RR inhibitors is directly correlated to RR activity levels [45], the hypersensitivity of the Δ*F4L* strain to HU suggests that these effects on DNA replication are caused by a reduction in RR activity.

### Δ*F4L* strains are uniquely hypersensitive to cidofovir and HU

We hypothesized that the impaired genome replication of the Δ*F4L* strain was due to reduced dNTP pool sizes as a result of decreased RR activity. However, it is difficult to interpret the meaning of biochemical measurements of pool sizes because of uncertainties surrounding how dNTPs are distributed in infected cells. Instead, we tested whether VACV RR mutants exhibit an altered sensitivity to the antiviral drug cidofovir (CDV). CDV is converted by cellular kinases to the diphosphoryl derivative (CDVpp) [46] which is competitive with respect to dCTP [47] and inhibits VACV E9 DNA polymerase activity [48], [49]. Thus, CDV sensitivity can be used as an indirect probe for changes in dCTP pool sizes. Table 2 summarizes how RR mutations affect CDV sensitivity as assessed by plaque reduction assays and calculated 50% effective concentration (EC_50_) values. Wild-type and Δ*F4L*/Δ*J2R* ^His*F4L*^ strains exhibited similar mean EC_50_ values of 42.0 and 41.2 µM, respectively. The Δ*I4L* strain was significantly more sensitive than the aforementioned strains (P<0.05) having a mean EC_50_ value of 25.1 µM. However, loss of *F4L* (or *F4L* and *J2R*) resulted in greater hypersensitivities to CDV (P<0.05) with EC_50_ values ∼5–7-fold lower than wild-type values. The Δ*F4L*/Δ*J2R* ^HisY300F*F4L*^ virus was even more sensitive to CDV (EC_50_ = 3.5 µM) than either wild-type (P<0.05) or Δ*F4L* (P<0.05) strains. As noted previously [50], [51], inactivation of *J2R* did not further alter VACV sensitivity to CDV (Table 2). The trends in CDV sensitivity closely mirrored those found in measurements of HU sensitivity using a plaque reduction assay (Table 2). The order of resistance to HU (from measurements of EC_50_) was wild-type ≥Δ*F4L*/Δ*J2R* ^His*F4L*^>Δ*I4L*>Δ*F4L*>Δ*F4L*/Δ*J2R* ^HisY300F*F4L*^ and seemed unaffected by the presence or absence of the *J2R* gene (Table 2). In order to determine if the hypersensitivities of Δ*F4L* and Δ*F4L*/Δ*J2R* ^HisY300F*F4L*^ strains to CDV and HU were specific and not simply due to the reduced replicative abilities of these viruses, we performed a plaque reduction assay using phosphonoacetic acid (PAA). PAA is a pyrophosphate analog and DNA polymerase inhibitor that is noncompetitive with dNTPs [52]. Therefore, the efficacy of PAA in inhibiting virus replication would not be expected to be dependent upon RR activity or dNTP pool sizes. Consistent with this, RR mutant VACV strains were not hypersensitive to PAA when compared to wild-type virus (Table 2). These mutant strains were also not hypersensitive to isatin-β-thiosemicarbazone (IBT), which causes aberrant late viral mRNA biogenesis [53] (data not shown). Collectively, these data all point to a deficiency in dNTP pools as being the cause of the Δ*F4L* strain growth deficiency (Figure 3) and suggest that F4, and not I4, is the critical determinant of growth efficiency and drug sensitivity.

**Table 2:** Susceptibility of VACV RR mutant strains to cidofovir (CDV), hydroxyurea (HU) and phosphonoacetic acid (PAA).

Virus	Mean EC_50_ of Compound					
	CDV (µM)^1^	Fold Change^2^	HU (mM)^1^	Fold Change^2^	PAA (µg/mL)^1^	Fold Change^2^
Wild-type	42.0 (36.2–48.7)	1.0	0.87 (0.72–1.06)	1.0	50.5 (41.9–61.0)	1.0
Δ*I4L*	25.1 (22.0–28.7)	**1.7**	0.19 (0.15–0.24)	**4.6**	55.6 (44.9–68.9)	1.1
Δ*F4L*	6.2 (5.5–7.0)	**6.8**	0.05 (0.04–0.06)	**17.4**	56.6 (49.4–64.9)	1.1
Δ*I4L*/Δ*F4L*	6.8 (5.4–8.5)	**6.2**	0.05 (0.04–0.06)	**17.4**	54.7 (48.3–62.1)	1.1
Δ*I4L*/Δ*F4L*/Δ*J2R*	7.6 (6.7–8.5)	**5.5**	0.05 (0.05–0.06)	**17.4**	47.4 (39.7–56.6)	1.1
Δ*F4L*/Δ*J2R*	8.1 (6.6–9.9)	**5.2**	0.07 (0.06–0.08)	**12.4**	49.0 (40.9–58.6)	1.0
Δ*F4L*/Δ*J2R* ^His*F4L*^	41.2 (35.9–47.1)	1.0	0.68 (0.50–0.91)	1.3	46.8 (38.3–57.1)	1.1
Δ*F4L*/Δ*J2R* ^HisY300F*F4L*^	3.5 (3.0–4.2)	**12**	0.03 (0.03–0.03)	**29**	44.9 (39.0–51.8)	1.1

1 Values in parentheses represent 95% confidence intervals.

2 Compared to mean EC_50_ of wild-type virus. Bold values indicate statistically significant (P<0.05) differences from wild-type values.

### Viral and human RR subunits interact in VACV-infected cells

**Figure 5:**
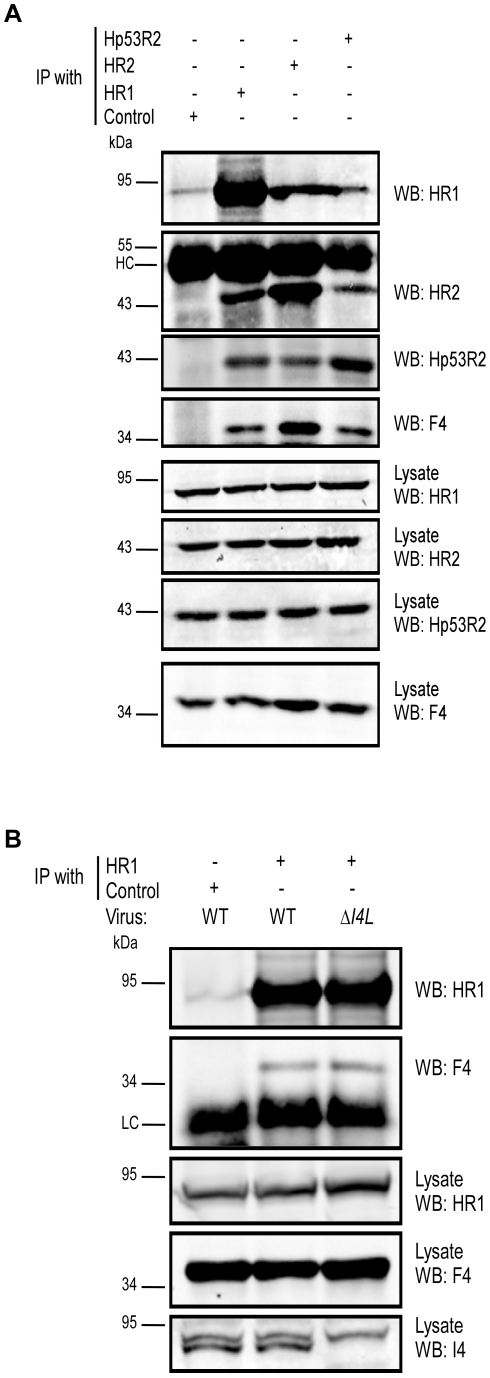
VACV F4 co-immunoprecipitates with endogenous human RR proteins. (A) Immunoprecipitation of HR1 in HeLa cells infected with wild-type VACV at a MOI of 10. At 6 h post-infection, cells were lysed and subjected to immunoprecipitation (IP) with antibodies directed against human R1 (HR1), human R2 (HR2) or human p53R2 (Hp53R2). Normal goat serum was used as a control. (B) Co-immunoprecipitation of F4 with HR1 in the presence or absence of I4. HeLa cells were infected with wild-type (WT) or Δ*I4L* VACV strains as in (A) and subjected to IP with HR1 or control antibodies 8 h post-infection. Western blots (WB) of IP material and total lysates are shown. LC, light chain; HC, heavy chain.

**Figure 6:**
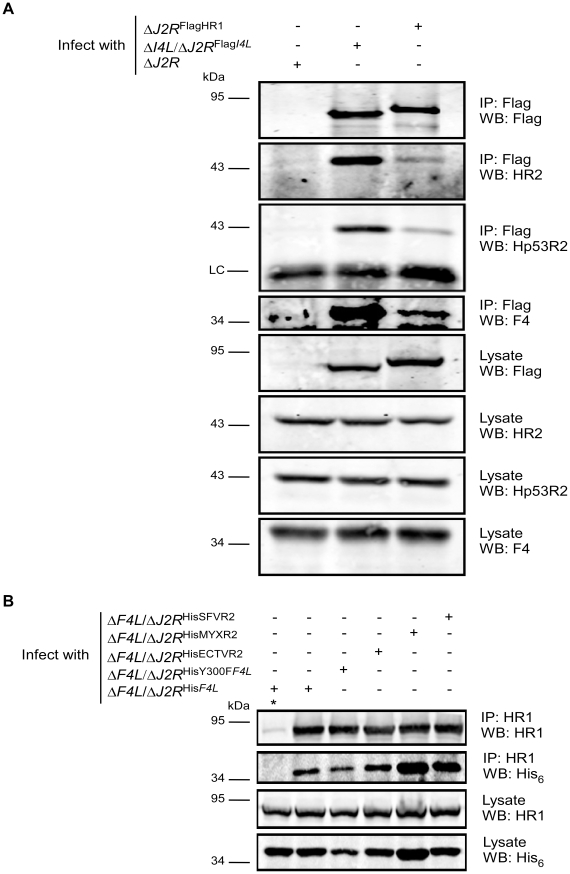
Recombinant poxvirus RR proteins interact with endogenous human RR proteins. (A) Co-immunoprecipitation of Recombinant Flag-tagged I4 and human R1 (HR1) with cellular and VACV RR proteins. HeLa cells were infected with the indicated strains at a MOI of 10 for 8 h after which protein extracts were prepared and immunoprecipitated (IP) and/or then western-blotted (WB) with the indicated antibodies. (B) Co-immunoprecipitation of VACV, ectromelia (ECTV), myxoma (MYX) and Shope fibroma (SFV) His_6_-tagged R2 proteins with HR1. HeLa cells were infected with the indicated strains at a MOI of 10 for 8 h and then protein extracts were subjected to immunoprecipitation with anti-HR1 antibodies or control serum (indicated by “*”). LC, light chain.

Our data suggested that F4 may form functional complexes with host R1 proteins to support viral replication. This hypothesis was strengthened by previous studies that found purified mouse and VACV RR subunits to form functional chimeric RR complexes *in vitro* [33]. To determine if virus-host RR interactions could occur *in vivo*, co-immunoprecipitation experiments were performed with VACV-infected HeLa cell lysates using antibodies against HR1, Human R2 (HR2) or Hp53R2 RR subunits. F4 co-immunoprecipitated with each of the host RR subunits (Figure 5A) and the efficiency of “pull-down” was the same in extracts prepared from cells infected with wild-type and Δ*I4L* strains (Figure 5B), suggesting that the presence of I4 does not significantly impede F4 interaction with host RR subunits. Interaction of F4 with cellular R2 subunits, while unexpected, may not be that surprising given that R2 subunits interact with one another in addition to interacting with homodimers of R1 [1]. We thought these interactions may be in part due to enhanced cellular RR subunit expression after infection. However, we were unable to observe induction of cellular RR expression by 24 h post-infection (Figure S2). To further confirm the immunoprecipitation results, VACV strains expressing either Flag-tagged HR1 (Δ*J2R* ^FlagHR1^) or Flag-tagged I4 (Δ*I4L*/Δ*J2R* ^Flag*I4L*^) were constructed and used in new immunoprecipitation experiments. Immunoprecipitation with anti-Flag antibodies confirmed the interaction of HR1 and I4 with F4 as well as with HR2 and Hp53R2 (Figure 6A). We typically observed weaker R2 bands in immunoprecipitations of Flag-tagged HR1 compared to Flag-tagged I4 despite similar amounts of these two proteins being immunoprecipitated (Figure 6A). This result was likely due to competition between the Flag-tagged HR1 protein and endogenous HR1, whereas Flag-tagged I4 is expressed in the Δ*I4L* background and thus does not have to compete for binding to R2 proteins with endogenous I4. We also prepared extracts from cells infected with Δ*F4L*/Δ*J2R* ^HisY300F*F4L*^ or Δ*F4L*/Δ*J2R* ^His*F4L*^ viruses and observed that these His_6_-tagged proteins could also be co-immunoprecipitated with HR1 protein (Figure 6B). Reciprocal co-immunoprecipitation experiments confirmed an interaction between F4 and HR1 proteins (Figure S3).

Other *Chordopoxvirus* R2 proteins rescued the replication defect of VACV Δ*F4L* strains (Figure 3B). Therefore, we determined whether these proteins could also interact with HR1. ECTV, MYXV, and SFV R2 proteins all co-immunoprecipitated with HR1 (Figure 6B). Although there appeared to be differences in the efficiency of HR1 association, western blotting of lysates showed that this reflected differences in R2 expression levels (Figure 6B). These results confirm that RR subunits from poxviruses that infect a diversity of mammalian hosts have conserved the capacity to interact with HR1.

In uninfected cells, mammalian RR subunits show an exclusively cytoplasmic distribution [54], [55], [56]. Confocal microscopy studies with antibodies directed against endogenous (Figure S4A) or epitope-tagged (Figure S4B) RR subunits suggested that VACV infection did not alter host RR localization and VACV RR subunits were also found to exhibit a similar cytoplasmic distribution.

### Requirement of C-terminal residues of F4 for interaction with HR1

**Figure 7:**
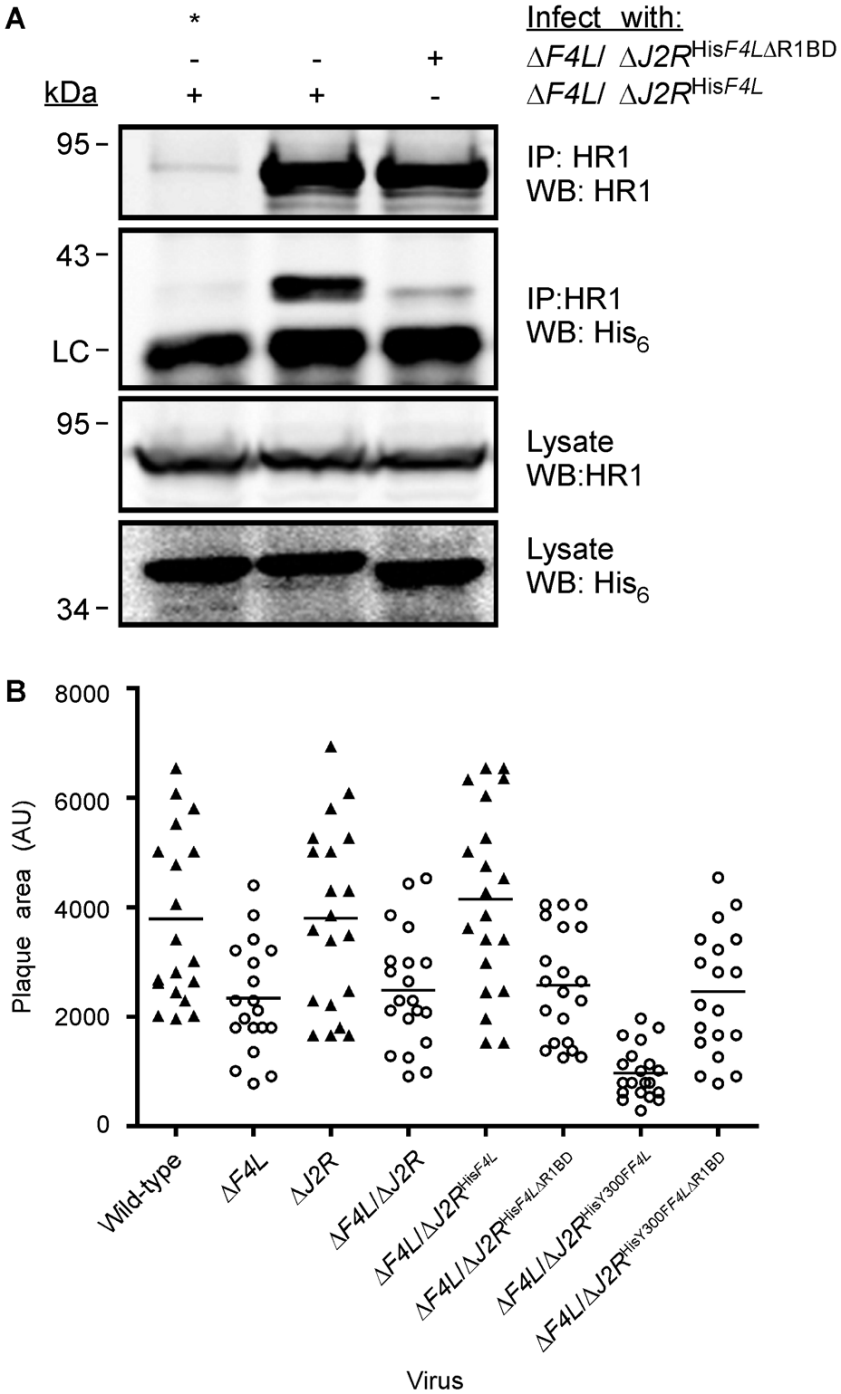
Deletion of F4 C-terminus residues inhibits interaction with HR1 and impairs virus growth. (A) Co-immunoprecipitation of recombinant F4 proteins with HR1. HeLa cells were infected with the indicated strains at a MOI of 10 for 8 h and then protein extracts were subjected to immunoprecipitation (IP) with anti-HR1 antibodies or control serum (indicated by “*”). Western blots (WB) of IP material and total lysates are shown. LC, light chain. (B) Plaque area analysis of RR mutant strains. BSC-40 monolayers in 60-mm-diameter plates were infected with ∼100 PFU of the indicated strains and stained 48 h post-infection with crystal violet. The scatter plots illustrate independent (n = 20) as well as mean (horizontal bar) plaque area measurements in arbitrary units (AU) for each of the indicated strains. Open circles indicate that the mean plaque area was statistically different (P<0.05) from wild-type virus as determined by a one-way ANOVA.

The previous studies showed that F4 interacts with HR1 but did not prove whether such an interaction was essential for viral replication. Numerous structural and peptide-inhibition studies of class I RR proteins have identified a C-terminal peptide (boxed in Figure 1) in R2 subunits as critical for interaction with R1 proteins [11], [57], [58], [59], [60], [61]. Since this C-terminal peptide is well conserved in F4 (Figure 1), we speculated that HR1-F4 interactions were also dependent on this peptide. To test this hypothesis, we generated the VACV strain Δ*F4L*/Δ*J2R* ^His*F4L*ΔR1BD^, encoding a truncation mutant of F4 that lacks the C-terminal seven residues representing the putative R1-binding domain (R1BD). We also generated an R1BD mutant that also encodes the Y300F substitution, (Δ*F4L*/Δ*J2R* ^HisY300F*F4L*ΔR1BD^). As shown in Figure 7A, His_6_-tagged F4 co-immunoprecipitated with HR1 in HeLa cell extracts. However, there was a clear reduction (by ∼90%) in co-immunoprecipitation of His_6_-tagged F4 proteins lacking the R1BD, despite comparable levels of these two forms of F4 in lysates and immunoprecipitates. Thus, F4 appears to have conserved the R1-binding peptide encoded by class I RRs.

We used plaque area measurements to determine if deleting the R1BD would alter VACV plating properties (Figure 7B). The control viruses exhibited the same relative plaque sizes noted previously (*i.e.* wild-type = Δ*F4L*/Δ*J2R* ^His*F4L*^>ΔF4L>Δ*F4L*/Δ*J2R* ^HisY300F*F4L*^) and the differences were all significant (P<0.05). However, the Δ*F4L*/Δ*J2R* ^His*F4L*ΔR1BD^ and Δ*F4L*/Δ*J2R* ^HisY300F*F4L*ΔR1BD^ strains produced plaques no different in size from those produced by Δ*F4L* strains (P>0.05). This suggested that the F4 R1BD was not only required for RR activity, but that the HR1-F4 interaction was also responsible for the dominant negative effects observed with strains encoding the Y300F-substituted F4 protein with an intact R1BD. We also confirmed in these studies that inactivation of *J2R* alone had no significant effect on plaque size (Figure 7B).

### Correlation of Δ*F4L* strain replication with host RR subunit expression

**Figure 8:**
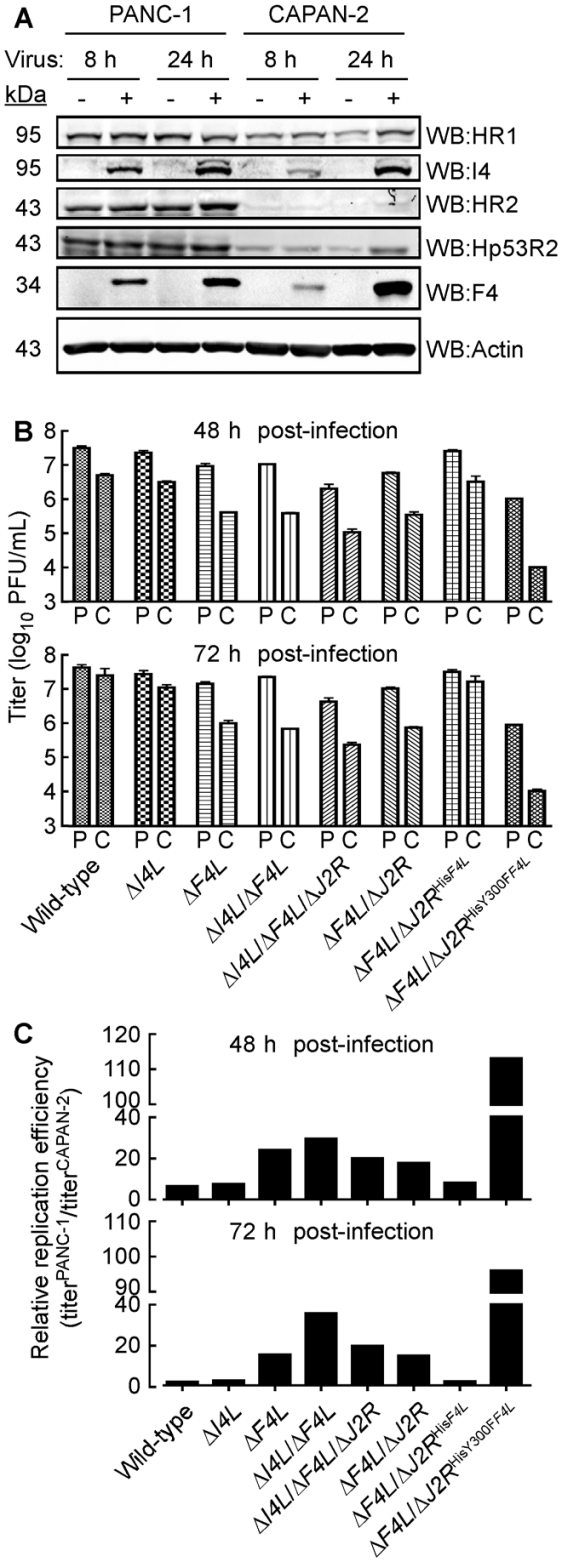
Correlation of cellular RR expression and VACV replication in two human cancer cell lines. (A) Western blot (WB) analysis of viral and cellular RR subunit expression. Protein extracts were prepared from mock-infected and wild-type-infected (MOI of 5) PANC-1 and CAPAN-2 cells at the indicated times post-infection. Western blots were then performed using antibodies with the indicated specificities. (B) Mean virus yields (+SE) after 48 or 72 h of infection (MOI of 0.03) of PANC-1 (P) or Capan-2 (C) cells with each of the indicated strains. (C) Re-plotting of the data in (B) to show the relative difference in mean replication efficiencies between the two cell lines for each strain. Relative differences were obtained by dividing yields obtained on PANC-1 cells by those obtained on CAPAN-2 cells.

Our results suggested that deleting the *F4L* gene renders VACV highly dependent upon the host cell for provision of a complementing RR activity. This leads to the prediction that the efficiency of growth of a Δ*F4L* virus will depend upon the level of cellular RR activity. To test this hypothesis, we used two pancreatic cancer cell lines that have been previously reported to exhibit high (PANC-1) and low (CAPAN-2) levels of RR subunit expression and activity [45], [62]. We prepared cell-free extracts from wild-type virus-infected (or mock-infected) PANC-1 and CAPAN-2 cells, and used western blots to measure the levels of RR proteins. This study confirmed that HR1, HR2, and Hp53R2 are expressed at lower levels in CAPAN-2 cells, relative to PANC-1 cells, and that this phenotype is unaffected by VACV infection (Figure 8A). We then seeded approximately equal numbers of PANC-1 and CAPAN-2 cells into culture dishes and infected them with wild-type and mutant strains. The total titers for each of these infections at 48 or 72 h post-infection are plotted in Figure 8B. Division of the mean titers obtained in PANC-1 cells by those obtained in CAPAN-2 cultures for each virus gave an estimate of the fold difference in replication efficiencies for each strain in these cells (Figure 8C). These data showed that PANC-1 cells support a relatively normal level of replication of most mutant VACV strains. For example, the wild-type virus grew only ∼3–6-fold better on PANC-1 cells than did Δ*F4L*, and Δ*I4L*/Δ*F4L*, and Δ*F4L*/Δ*J2R* strains (Figure 8B). One exception to this rule is that the wild-type virus produced titers ∼16-fold higher than the Δ*I4L*/Δ*F4L*/Δ*J2R* strain on PANC-1 cells (Figure 8B). This suggested that in certain cell types and in the absence of *J2R* and *F4L*, *I4L* may play some role in supporting VAC replication.

A more notable feature of this experiment is that all virus tested grew better on PANC-1 cells compared to CAPAN-2 cells. The wild-type, Δ*I4L*, and Δ*F4L*/Δ*J2R* ^His*F4L*^ strains produced yields 6–8-fold higher on PANC-1 cells than CAPAN-2 cells 48 h post-infection and this difference was greatly exacerbated by deletion or mutation of *F4L* (Figure 8C). For example, the Δ*F4L* strain grew 18–30-fold better on PANC-1 cells and the Δ*F4L*/Δ*J2R* ^HisY300F*F4L*^ strain yielded a 113-fold increase in titer on PANC-1 cells compared to CAPAN-2 cells. In fact, titering of input inocula indicated that the Δ*F4L*/Δ*J2R* ^HisY300F*F4L*^ strain did not productively replicate in CAPAN-2 cells (data not shown). This suggested that the reduced RR activity of CAPAN-2 cells imposes a barrier to replication of this mutant. Collectively, these results suggested that the replication defects exhibited by Δ*F4L* and Δ*F4L*/Δ*J2R* ^HisY300F*F4L*^ strains can be complemented in human cancer cell lines over-expressing cellular RR subunits. However, direct evidence for the linkage between cellular RR levels and mutant rescue requires further studies.

### VACV RR subunits are differentially required for pathogenesis in mice

**Figure 9:**
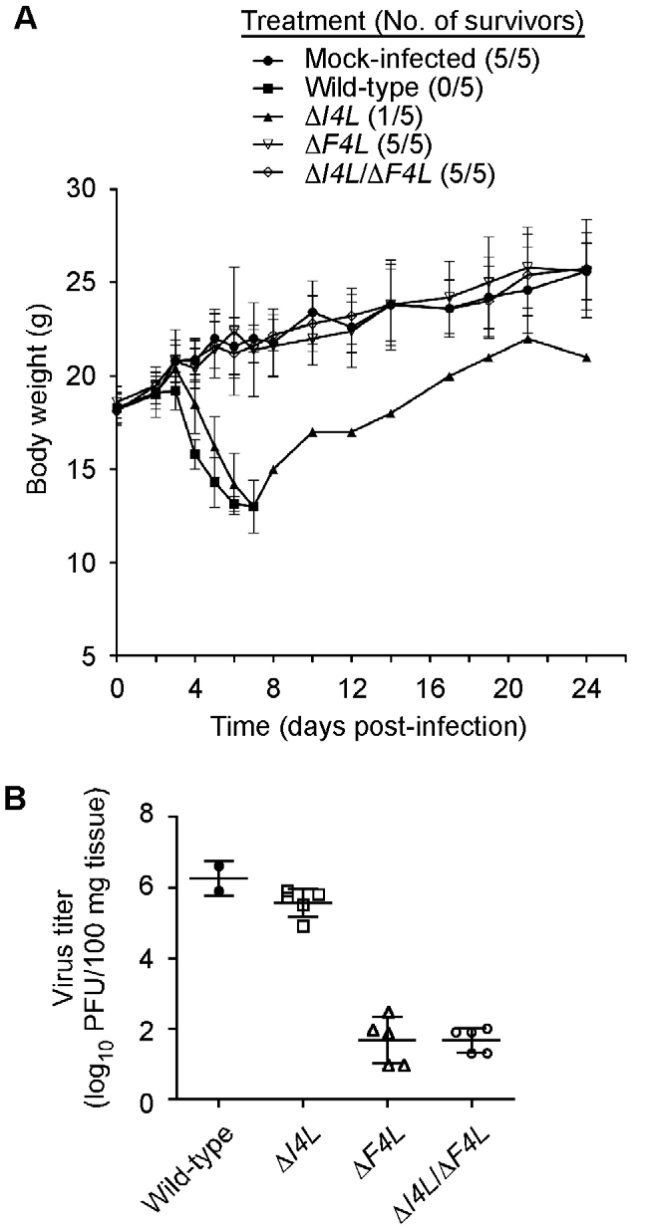
Differential requirement of VACV RR subunits for pathogenesis. (A) Analysis of animal body weight after infection with RR mutant strains. Groups of 5 NMRI mice were inoculated by an intranasal route with 40,000 PFU of the indicated VACV strains or were mock-infected with sterile buffer. Symbols represent mean body weight of each group of mice (or surviving members) over the indicated times post-infection. The number of surviving mice in each treatment group is indicated in parentheses. Error bars represent SD. (B) Lung titers after infection with RR mutant strains. The scatter plot shows lung virus titers from individual mice with means (horizontal bars) for each group. Mice were infected in parallel with studies in (A) and were euthanized 5 days post-infection. Lung virus titers were determined as described in Materials and Methods.

We used an animal model to determine if the apparent differential requirement for VACV RR subunits for replication in culture would be recapitulated *in vivo*. We infected groups of five NMRI mice with equal doses of wild-type, Δ*I4L*, Δ*F4L*, or Δ*I4L*/Δ*F4L* strains and tracked changes in animal body weight over 24 days. The wild-type and Δ*I4L* strains exhibited a similar degree of virulence, causing the death of 5/5 and 4/5 animals, respectively, within seven days of infection. In contrast, both Δ*F4L* and Δ*I4L*/Δ*F4L* strains were highly attenuated, with all animals displaying little to no signs of disease and surviving the infections (Figure 9A). There were small, transient drops in body weight for animals infected with the Δ*F4L* strain around days 5 and 7, otherwise these animals, and those infected with the Δ*I4L*/Δ*F4L* strain, showed no obvious signs of morbidity when compared to the mock-infected control group (Figure 9A). To obtain a more quantitative measurement of the pathogenic nature of these infections, we isolated lung tissues from mice infected with the aforementioned strains on day 5 post-infection. Wild-type and Δ*I4L* strains clearly had a replication advantage over Δ*F4L* and Δ*I4L*/Δ*F4L* strains with lung titers approximately 4 logs higher than the latter two strains (Figure 9B). These results indicate that VACV RR subunits are differentially required for virulence in mice.

## Discussion

Acquisition of a suitable supply of dNTPs to support replication is a challenging feat for mammalian DNA viruses because most host cells exist predominantly in a terminally-differentiated and quiescent state [63]. The S-phase-specific nature of host R2 expression leaves quiescent cells with only p53R2-R1 complexes to maintain a low (∼2–3% the level of cycling cells [64]) level of RR activity to meet the demands of DNA repair and mitochondrial genome synthesis [16], [19], [65]. Since ribonucleotide reduction is the rate-limiting step in mammalian dNTP biogenesis [26], low RR activity may pose a barrier to productive infection. Therefore, DNA viruses must replicate only in cycling cells, induce host RR activity upon infection, and/or encode their own RR enzymes [63]. Many large DNA viruses, including herpes-, irido-, asfra- and poxviruses have evolved the later strategy.

It is clear that herpesvirus-encoded RR proteins are important because inactivation of viral RR genes leads to replication defects *in vitro* and in animals [66], [67], [68], [69]. Furthermore, inhibiting complex formation by herpes simplex virus (HSV) R1 and R2 proteins with a C-terminal R2 peptide mimic has been shown to prevent HSV replication in culture [70], [71], [72]. Interestingly, β-herpesviruses, only encode an R1 gene, although it is still required for virulence [63]. However, it is unlikely to play a catalytic role in dNTP biogenesis as it encodes mutations at key catalytic residues that would render this subunit inactive in RR complexes [73], [74], [75]. Recent evidence suggests that β-herpesviruses may induce host RR protein expression, possibly explaining why viral RR function was not conserved [75], [76]. What biological purpose is served by β-herpesvirus R1 proteins is unclear, although it has been suggested that these proteins might play some role in inhibiting apoptosis [77].

The increasing availability of virus genome sequences has revealed that differential conservation of viral RR genes is actually a widespread phenomenon among eukaryotic DNA viruses. However, in contrast to the case of β-herpesviruses, most DNA viruses that encode a single RR subunit encode R2 proteins while R1 is frequently absent. For example, iridoviruses in the *Megalocytivirus* genus only encode an R2 subunit while all other iridoviruses encode both RR subunits [78]. Furthermore, certain members of the *Phycodnaviridae* and *Ascoviridae* viral families also only encode R2 subunits [79]. This R2 bias is also seen in bacteriophage belonging to the *Siphoviridae* and *Myoviridae* families, suggesting that even prokaryotic viruses have biased conservation of RR genes [79].

Perhaps the most biased conservation of RR genes is found in poxviruses, with a clear favoring of R2 over R1 (Table S1). Even Orthopoxviruses, which typically encode both R1 and R2 genes, contain a member (horsepox virus) that encodes a fragmented R1 gene [80]. Many other Chordopoxviruses show no evidence of ever having encoded an R1 activity, including the Leporipoxviruses MYXV and SFV which we sequenced ten years ago [81], [82]. At that time, the close similarity of MYXV and SFV R2 subunits to mammalian R2 proteins, and absence of a viral R1 homolog, led us to suggest that *Leporipoxvirus* R2 subunits were likely forming chimeric complexes with host R1 proteins [82]. Subsequent biochemical studies of VACV F4 and I4 by Chimploy and Mathews [33] found that mixing purified F4 and I4 with MR1 and MR2 proteins, respectively, resulted in functional, chimeric RR enzymes. However, the two kinds of chimeric RRs did not display identical properties. Whereas the native complexes (*i.e.* I4_2_F4_2_ and MR1_2_MR2_2_) were about equally active, the I4_2_MR2_2_ enzyme exhibited ∼5-fold less activity and the MR1_2_F4_2_ enzyme showed up to 2-fold *more* activity than either native complex [33]. These observations may explain why Child *et al.* reported their Δ*I4L* strain to exhibit no observable replication defect in culture and only a small (∼10-fold) increase in LD_50_ for mice compared to wild-type VACV [34]. However, in an attempt to develop new vaccine strains, Lee *et al.* generated a *F4L* insertional inactivation mutant and reported significant increases of ∼1000-fold in the LD_50_ of this mutant in a similar mouse model used by Child *et al.* with their Δ*I4L* strain [34]. Collectively these independent bioinformatic, biochemical, and molecular genetic studies all suggested that poxvirus R2 subunits might be more important for viral replication than R1 subunits. However, the contributions of poxvirus R1 and R2 subunits to viral replication and pathogenesis had never been directly compared nor was it clear why R2 subunits may be more important to the poxvirus life cycle.

We examined this issue in detail by generating a panel of VACV RR mutant strains and analyzing their plaque, growth, and pathogenic properties. Our studies clearly show that these properties are far more affected in Δ*F4L* strains compared to Δ*I4L* strains (Figures 3 and 9). Combining *F4L* and *I4L* deficiencies caused no further inhibition of virus growth, suggesting that the phenotype is dominated by the integrity of the *F4L* locus. We also showed that inactivating VACV *J2R* in the Δ*F4L* background did not further impede replication (Figure 3), suggesting that the salvage pathway for dNTP production is either not required for replication in culture, or is sufficiently complemented by host TK enzymes. This result made it possible to use the *J2R* locus as a site for introducing ectopic copies of different recombinant R2 proteins. The Δ*F4L* strain's phenotype can be completely complemented by a gene encoding His_6_-tagged F4 and by genes encoding other *Orthopoxvirus* or *Leporipoxvirus* R2 proteins (Figure 3B). Why Hp53R2 failed to rescue this phenotype is unclear but several possibilities exist. For one, R1-p53R2 complexes exhibit only 40–60% the activity of R1-R2 complexes [17] and this reduced activity might not meet some activity threshold required for efficient viral replication. Secondly, significant fractions of Hp53R2 proteins are bound in inactive complexes by p53 and p21 proteins, and are only released after appropriate signaling pathways have been activated [83], [84]. Therefore, even if one over-expresses Hp53R2, it may not produce a sufficient level of “free” Hp53R2 that could complex with R1 proteins. Finally, Hp53R2 has recently been shown to inhibit MEK2, a kinase involved in the activation of the Ras-Raf-MAPK signaling pathway [85]. This inhibition could be detrimental as activation of the MAPK pathway is required for VACV replication [86]. These possibilities are currently being addressed. Attempts to generate a VACV strain over-expressing HR2 have thus far been unsuccessful and so it is unclear whether HR2 can complement a Δ*F4L* strain.

The hypothesis that F4 protein can compete with (or replace) cellular small subunits to form chimeric RR complexes *in vivo* is strongly supported by the dominant-negative phenotype exhibited by Y300F-substituted F4 protein (Figure 3). Viruses encoding these mutant proteins replicate very poorly and produce extremely small plaques. Furthermore, this phenotype is not altered by the presence or absence of I4 (Figure 3B). The genetic data are fully concordant with our immunoprecipitation experiments, which showed that F4 interacted with cellular RR subunits (Figure 5A) and that this interaction was unaffected by I4 (Figure 5B). We also found that other *Chordopoxvirus* R2 proteins co-immunoprecipitated with HR1 (Figure 6B), which is consistent with the ability of these proteins to rescue the replication defect of the Δ*F4L* strain. The ability of various poxvirus R2 subunits to interact with HR1 might be explained by the high degree of sequence conservation amongst mammalian RR subunits and the fact that *Chordopoxvirus* R2 subunits are typically >70% identical to mammalian subunits. The observation that poxvirus RR genes are generally more similar to cellular RR genes than other virus RR genes has led to the suggestion that poxviruses have acquired RR genes through horizontal transfer events with their host [87], [88]. Interestingly, other viral and bacterial pathogens have also likely acquired RR enzymes through host gene capture [89], [90], [91]. It would be of interest to determine if *Chordopoxvirus* R2 proteins exhibit a quantitative binding preference for R1 proteins isolated from their natural hosts (*e.g.* MR1 with ECTV R2 and rabbit R1 with MYXV and SFV R2), as that would be an expected consequence of evolutionary adaption to a particular host. Potential differences in binding affinities between poxvirus and host subunits may provide further insight into factors that contribute to poxvirus host range which remain poorly defined.

During infection, ∼8-fold more F4 than I4 subunits are synthesized [92]. In tissue culture, levels of mammalian R1 are constant during the cell cycle due to its long half-life [13], while R2 subunits are quickly degraded late in mitosis leading to a much shorter half-life [15]. Given the relatively reduced activity of R1-Hp53R2 complexes [17], it is possible that production of F4 in excess allows these subunits to form needed complexes with both viral and host R1 subunits. Interestingly, poxvirus R2 subunits, like Hp53R2, lack much of the N-terminal sequences found in HR2 including phosphorylation and ubiquitination sites that may regulate HR2 function and degradation (Figure 1) [15]. This may explain why F4 protein levels are stable for at least 12 h after infection [92]. It seems likely that adaptive changes during evolution has led to conservation of poxvirus R2 enzymatic function yet has resulted in a loss of regulatory sequences that may restrict viral subunit levels in the host.

We hypothesized that the ability of *Chordopoxvirus* R2 proteins to interact with HR1 was due to the high degree of conservation of the C-terminal seven residues between poxvirus and mammalian R2 subunits (Figure 1). This C-terminal motif has been well-characterized in R1-R2 interaction studies of various class I RR enzymes [11], [57], [58], [59], [60], [61] and an oligopeptide mimic (^7^FTLDADF^1^) of mammalian R2 C-termini has been shown to inhibit RR activity [57]. Positions 1, 5, and 7 in this mimic are the most critical determinants of RR inhibition [57], and the residues at these positions are conserved in the C-terminus of F4 (FSLDVDF) suggesting that VACV and mammalian RR share a common R1-R2 subunit interaction mechanism. The large differences between C-terminal sequences of HSV (YAGAVVNDL) and mammalian R2 subunits likely explains why no evidence could be found for interaction of HSV RR proteins with host subunits [23] and why peptide mimics of the HSV R2 C-terminus are highly selective antivirals [71]. Previous studies have used the F4 heptapeptide to generate an affinity column for I4 purification [93]. Therefore, we thought it was likely that F4 interacted with R1 proteins in a similar manner as found with cellular R2 subunits. Indeed, interaction of F4 proteins lacking the putative R1BD with HR1 was clearly impaired (Figure 7A) and strains expressing these truncated proteins were unable to rescue the small plaque phenotype of the Δ*F4L* mutant (Figure 7B). However, deleting the R1BD from Y300F F4 did suppress the dominant negative phenotype (Figure 7B), which further implied that F4 functionally interacts with HR1 through the C-terminus of F4.

Collectively our data show that VACV F4 proteins (and likely other poxvirus R2 proteins) are required for efficient viral replication in culture as well as for pathogenesis (Figure 9). While our studies of CDV and HU sensitivities (Table 2) suggest a defect in RR activity and subsequent dNTP pool biogenesis as the underlying cause for the defect of Δ*F4L* strains, it is possible that these are only indirect consequences of inactivation of *F4L* and other functions of F4 are required for replication. However, the dominant negative phenotype of the Y300F-encoding strains in the presence or absence of I4 (Figure 3B), the requirement of the R1BD to produce this phenotype and interact with HR1, and the similar localization observed with viral and cellular RR subunits in infected cells (Figure S4) all support the simple conclusion that F4 must form complexes with host R1 proteins to facilitate dNTP biogenesis.

The critical importance of this interaction for VACV replication suggests that Δ*F4L* strains may act as selective oncolytic agents. A wide variety of human cancers exhibit elevated RR expression patterns and prolonged treatment of patients with RR inhibitors can lead to drug resistance as result of HR2 gene amplification [94], [95], [96]. For example, a recent study of patients with non-small lung cancer found that elevated host RR expression levels in patients' tumors were directly correlated with reduced response to chemotherapy and poorer prognoses [94]. Interestingly, hrR3, a HSV mutant strain with an inactivated viral R1 gene, replicates more efficiently in cancers with elevated host RR expression [97] and shows promise as an oncolytic agent in mouse models [98]. The enhanced replication of Δ*F4L* and Δ*F4L*/Δ*J2R* ^HisY300F*F4L*^ strains in PANC-1 cells relative to CAPAN-2 pancreatic cancer cell lines (Figure 8C) correlates well with the higher levels of RR subunits in PANC-1 cells (Figure 8A) and documented differences in RR activities between these two cell lines [45]. While further evidence will be needed to prove that host RR proteins complement the Δ*F4L* strain replication defect in PANC-1 cells, our results build a strong circumstantial case for the dependence of these *F4L* mutant strains on host RR activity. Since VACV and other, non-Orthopoxviruses (*e.g.* Leporipoxviruses [99], [100], and Yatapoxviruses [101]) that encode R2 subunits have shown potential for use in cancer virotherapy, we suggest that deletion of R2 subunit genes from these viruses may create more selective oncolytic agents.

During our studies we noted that poxviruses that encode R2 and TK genes tend to have higher A+T base content in their genomes than poxviruses lacking these genes (Table S1). This strong correlation suggests that hybrid virus-host RR complexes and/or poxvirus TK proteins have contributed to the establishment of unique dNTP pools that have influenced viral genome composition during the co-evolution of poxviruses with their hosts. Recently an APC mimic has been identified in poxviruses that lack R2 and TK genes including Molluscipoxviruses, Parapoxviruses, and crocodilepox virus [102]. The APC mimic in orf virus, termed “PACR” (poxvirus APC/cyclosome regulator) inhibits APC activity and causes mammalian cells to accumulate in G2/M phases of the cell cycle [102]. Since APC targets mammalian TK [103] and R2 [15] proteins for degradation during mitosis, poxvirus APC mimics may serve to prevent degradation of these host nucleotide metabolism proteins during viral replication. This is supported by the finding that PACR inhibits host TK degradation during the cell cycle [102]. This reliance on host nucleotide metabolism proteins may explain why poxviruses encoding APC mimics have low A+T content in their genomes (Table S1). Strict reliance on host nucleotide metabolism machinery may also explain why GC-rich Mollusci- and Parapoxviruses have a rather limited host range when compared to AT-rich Orthopoxviruses which encode their own RR and TK [104] (Table S1). Therefore, poxviruses appear to have acquired different mechanisms to obtain dNTPs for replication, which may ultimately influence their genomic composition and host tropism.

## Materials and Methods

### Cell and virus culture

Cell and virus culture methods have been described elsewhere [105]. Wild-type VACV and its mutant derivatives were derived from strain Western Reserve (WR) originally acquired from the American Type Culture Collection. Non-transformed African Green Monkey kidney cells (BSC-40) were normally cultured in modified Eagle's medium (MEM) supplemented with 5% fetal bovine serum (FBS). HeLa human cervical adenocarcinoma and human embryonic lung (HEL) cells were cultured in Dulbeccos MEM (DMEM) supplemented with 10% FBS. PANC-1 and CAPAN-2 cells are human pancreatic epithelioid carcinoma and adenocarcinoma lines, respectively and were also cultured in DMEM supplemented with 10% FBS. All of the above cell lines were originally obtained from the American Type Culture Collection. A U20S human osteosarcoma cell line that expresses Cre recombinase was a kind gift from Dr. J. Bell (University of Ottawa). These cells were maintained in DMEM supplemented with 10% FBS. Cells were cultured in Opti-MEM media (Invitrogen; Carlsbad, CA) for experiments requiring transfections.

### Materials

(*S*)-1-[3-hydroxy-2-(phosphonomethoxy)propyl]cytosine or cidofovir (CDV or HPMPC) was from Dr. K. Hostetler (University of California, San Diego). Hydroxyurea (HU) was obtained from Alfa Aesar (Ward Hill, MA). X-gal and X-glu substrates were obtained from Sigma Chemical Co. (St. Louis, MO) and Clontech (Palo Alto, CA), respectively. Phosphonoacetic acid (PAA) was from Sigma Chemical Co. Isatin-β-thiosemicarbazone (IBT) was from Pfaltz and Bauer (Waterbury, CT). Mycophenolic acid (MPA) and xanthine were obtained from Sigma Chemical Co. Hypoxanthine was obtained from ICN Biomedicals, Inc. (Aurora, OH). Compounds were diluted to their final concentration in MEM (CDV; HU; PAA; IBT) or in a 1∶1 mixture of MEM and 1.7% noble agar (X-gal; X-glu) immediately prior to use. *Taq* and *PfuUltra* DNA polymerases were obtained from Fermentas (Burlington, ON) and Stratagene (La Jolla, CA), respectively.

### Antibodies, western blotting, and immunoprecipitation

Normal mouse and goat serum and goat polyclonal antibodies against human R1 (HR1), human R2 (HR2), and human p53R2 (Hp53R2) were from Santa Cruz Biotechnology, Inc. (Santa Cruz, CA). Mouse monoclonal antibodies against HR1 and HR2 were from Millipore (Billerica, MA) and Santa Cruz Biotechnology, Inc., respectively. Mouse monoclonal antibodies against Flag and His_6_ (His) epitopes were from Sigma and Roche (Mississauga, ON), respectively. Rabbit anti-Flag epitope polyclonal antibodies were obtained from Sigma. A mouse monoclonal antibody was raised against bacterially-expressed, recombinant ECTV R2 antigen by ProSci (Poway, CA). The resulting antibody also recognizes VACV F4 and was used for western blotting. In some cases, a rabbit anti-F4 polyclonal antibody was also used for western blotting. The plasmid used to express recombinant ECTV R2 antigen and the rabbit anti-F4 antibody were kindly provided by Dr. M. Barry (University of Alberta). A rabbit anti-VACV I4 polyclonal antibody was obtained from Dr. C. Mathews (Oregon State University). Although this antibody recognizes VACV I4, it also cross-reacts with cellular R1 on western blots [92]. The mouse monoclonal antibody against VACV I3 has been described [40] and the mouse monoclonal antibody against cellular actin was from Sigma.

Protein extracts for western blots and immunoprecipitations were prepared from cell cultures by lysing cells on ice in a buffer containing 150 mM NaCl, 20 mM Tris (pH 8.0), 1 mM EDTA, and 0.5% NP-40 along with freshly-added phenylmethylsulfonyl fluoride (100 µg/mL) and protease inhibitor tablets (Roche). For western blots, 20–40 µg of total protein were subjected to SDS-PAGE and subsequently blotted with appropriate antibodies after transfer to nitrocellulose membranes. Membranes were scanned using an Odyssey scanner (Li-COR Biosciences).

Protein extracts for immunoprecipitations were recovered as described above 6–8 h post-infection from 10^7^ HeLa cells infected with indicated strains at a multiplicity of infection (MOI) of 10. Extracts were then pre-cleared by incubation with normal mouse or goat serum along with protein G sepharose beads (GE Healthcare Life Sciences; Piscataway, NJ) for 30 min at 4°C with constant inversion. The samples were subsequently centrifuged (2,500 rpm, 1 min, 4°C) and supernatants were transferred to fresh tubes. These extracts were then incubated with the primary antibodies overnight at 4°C with constant inversion. Fresh protein G beads were then added to the extracts and incubated for 2 h at 4°C after which the beads were collected (2,500 rpm, 1 min, 4°C) and washed four times with lysis buffer. The resulting bead-protein complexes were resuspended in SDS-PAGE loading buffer, boiled for 15 min and subjected to SDS-PAGE. Western blotting was then performed as described above. Whole cell extracts (lysates) were also blotted with indicated antibodies and represented ∼5% of the input material used for immunoprecipitations.

### Plaque morphology and replication analyses

Plaque dimensions were measured on 60-mm-diameter dishes of confluent BSC-40 cells infected with ∼100 plaque-forming units (PFU) of the indicated strain. After 48 h of infection, triplicate plates were stained with crystal violet and scanned using an HP ScanJet 6300C scanner. The resulting image files were analyzed using ImageJ v1.04g software (National Institutes of Health, USA). Unpaired *t*-tests or one-way ANOVA tests were performed on mean plaque areas between wild-type and each of the various RR mutant strains using GraphPad Prism (San Diego, CA) software (version 4.0). In some cases two different RR mutant strains were also compared for differences in mean plaque areas. A P value of <0.05 was considered to be statistically significant.

Growth analyses were conducted in BSC-40, HeLa, PANC-1 and CAPAN-2 cell cultures using the indicated MOI and strains. Cells were harvested by scraping monolayers into the culture media at the indicated time points followed by three rounds of freeze-thawing. Virus stocks were titered on BSC-40 cells.

For viral genome replication analyses, at the indicated times post-infection, BSC-40 cells were harvested by scraping, collected by centrifugation (800 rpm, 10 min, 4°C) washed once with PBS, and resuspended in 500 µL of 10× saline-sodium citrate (SSC) loading buffer containing 1 M ammonium acetate [106]. The cells were then disrupted by three cycles of freeze-thaw and 50-µL aliquots of the lysates were applied to a Zeta probe membrane using a slot-blot apparatus (Bio-Rad, Richmond, CA). Samples were denatured with 1.5 M NaCl and 0.5 M NaOH and washed twice with 10× SSC loading buffer. The membrane was then hybridized with a ^32^P-labeled *E9L* gene probe. After the membrane was washed with SSC buffer and air dried, it was exposed to a phosphorimager screen, imaged using a Typhoon 8600 phosphorimager and the data were processed using ImageQuant software, (version 5.1) [40]. In some cases 0.5 mM HU was added to the media 1 h post-infection.

### Plaque reduction assays

Plaque-reduction assays were performed as previously described [105]. Briefly, 35-mm-diameter dishes of confluent BSC-40 cells were inoculated with ∼100 PFU of the indicated virus strains, and 1 h after infection either drug-free medium or medium containing the indicated doses of CDV or HU was added to the cultures and the plates were incubated at 37°C for 48 h. Plates were then stained with crystal violet to visualize and count plaques. Mean 50% effective concentration (EC_50_) values and their 95% confidence intervals (CIs) were calculated using nonlinear regression analyses with GraphPad Prism software after three independent experiments had been performed. In cases where the 95% CIs of two different EC_50_ values did not overlap, these two EC_50_ values were considered to be statistically different (P<0.05).

### Confocal microscopy

HeLa cells were grown on coverslips in 24-well plates and infected with the indicated virus strains at a MOI of 5 for 10 h. The cells were fixed for 30 min on ice with 4% paraformaldehyde in PBS. The fixed cells were blocked and permeabilized for 1 h at RT in PBS containing 0.1% Tween (PBS-T) as well as 10% BSA. The coverslips were then incubated with the primary antibodies diluted in PBS-T (1% BSA) for 2 h at RT, washed three times and then incubated with secondary antibodies conjugated to Alexa 488 or 594 (Invitrogen) for 1 h at RT. The cells were then counterstained with 10 ng/mL 4′,6′-diamidino-2-phenylindole (DAPI) in PBS-T for 15 min. The specimens were examined using a Zeiss 710 Laser-Scanning confocal microscope equipped with DAPI, Alexa 488, and Alexa 594 filters. Images were captured and processed using ZEN 2009 software and Adobe Photoshop (version 10.0.1).

### Recombinant viruses

BSC-40 cells were grown to confluence and then infected for 1 h with the appropriate VACV strain (see below) at a MOI of 2 in 0.5 mL of PBS. The cells were then transfected with 2 µg of linearized plasmid DNA using Lipofectamine 2000 (Invitrogen). See Text S1 and for details regarding the primers and transfer vectors used to generate recombinant VACV strains. The cells were returned to the incubator for another 5 h, the transfection solution was replaced with 5 mL of fresh growth medium, and the cells were cultured for 24–48 h at 37°C. Virus progeny were released by freeze-thawing, and the virus titer was determined on BSC-40 cells. To identify recombinant virus, plaques were stained with X-gal or X-glu (both at 0.4 mg/mL) in solid growth media, or cultured in media containing 25 µg/mL MPA supplemented with xanthine (250 µg/mL) and hypoxanthine (15 µg/mL) for selection of *yfp*-*gpt*-encoding strains (see Text S1; [107]). The PCR was used to confirm insertions/deletions in the resulting recombinant viruses. The primers: 5′-GATGAATGTCCTGGATTGGA-3′ & 5′-ATTCCAAAGATCCGACGGTA-3′ were used to PCR amplify ∼700 bp of *I4L* sequence that should not be present in Δ*I4L* strains. The primers: 5′-ATGGAACCCATCCTTGCACC-3′ & 5′-ATCTTCTTGAGACATAACTC-3′ were used to amplify ∼930 bp of *F4L* sequence that should not be present in Δ*F4L* strains. Disruption of *J2R* sequence was detected with primers: 5′-TCCTCTCTAGCTACCACCGCAATAG-3′ & 5′-GTGCGGCTACTATAACTTTTTTCC-3′ that bind to regions of *J2R* flanking the insertion site of pSC66 vector [108] sequences (see below). Primers TGGATTCGTACAAATTGGATTCTAT & AATTGCTATTTCAGAGATGAGGTTC were used to amplify an ∼800 bp fragment from VACV DNA polymerase (*E9L*) sequence to serve as a positive control for amplification. In some cases western blotting was used to confirm the presence or absence of gene expression in the described VACV strains. Details of how each VACV strain was constructed are provided in Text S1 and the marker rescue strategies used in these studies are depicted in Figure 2A.

*PfuUltra* DNA polymerase (Stratagene) was used to PCR-amplify DNA for cloning whereas *Taq* DNA polymerase (Fermentas) was used for PCR diagnostic purposes. Plasmid constructs were verified by sequencing and all virus strains were plaque-purified a minimum of three times in BSC-40 cells or Cre recombinase-expressing U20S cells. All VACV strains were characterized by PCR (data not shown) and sometimes by western blotting (Figure 2B), although for brevity only characterization of the main viral strains discussed throughout this study is shown.

### Animal studies

Female NMRI mice, 3 to 4 weeks of age, were obtained from Charles River Laboratories (Brussels, Belgium). Mice were utilized at 5 mice per infection or control group for morbidity studies. Mice were anesthetized using ketamine-xylazine and inoculated intransally (or mock-inoculated) with 4×10^4^ PFU of virus diluted in 30 µL of saline. Animal body weights were recorded over the next 24 days or until the animals had to be euthanized because of more than 30% loss in body weight. To determine viral titers in lungs, two (wild-type infections) or five animals (Δ*I4L*, Δ*F4L*, and Δ*I4L*/Δ*F4L* infections) were euthanized on day 5. Lung samples were removed aseptically, weighed, homogenized in MEM, and frozen at −70°C until assayed by titrations on HEL cells. For mouse pathogenicity experiments, the Δ*I4L*/Δ*F4L* strain was generated with the pDGloxPKO^DEL^ vector. This allowed for removal of the *yfp*-*gpt* marker cassette from the *I4L* locus after passage in U20S cells expressing Cre recombinase. No differences were found in replication in culture between strains expressing the *yfp*-*gpt* cassette and those with this cassette deleted by Cre recombination (Figure S5). See Text S1 for further details.

### Ethics statement

All animal work was approved by the K.U. Leuven Animal Care and Use Committee. All animal guidelines and policies were in accordance with the Belgian Royal Decree of 14 November 1993 concerning the protection of laboratory animals and the European Directive 86-609-EEC for the protection of vertebrate animals used for experimental and other scientific purposes.

### Genbank gene ID numbers

1. VACV *I4L* (VACWR073) (Genbank ID: 3707606)
2. VACV *F4L* (VACWR043) (Genbank ID: 3707500)
3. VACV *J2R* (VACWR094) (Genbank ID: 3707550)

### Genbank protein accession numbers

1. human R2 (HR2; Genbank accession: NP_001025.1)
2. mouse R2 (MR2; Genbank accession: NP_033130.1)
3. human p53R2 (Hp53R2; Genbank accession: BAD12267.1)
4. mouse p53R2 (Mp53R2; Genbank accession: Q6PEE3.1)
5. VACV F4 (Genbank accession: AAO89322.1),
6. ectromelia EVM028 (Genbank accession: NP_671546.1),
7. myxoma virus m015L (Genbank accession: NP_051729.1),
8. Shope fibroma gp015L (Genbank accession: NP_051904.1)
9. human R1 (HR1; Genbank accession: AAD37491)
10. mouse R1 (mR1; Genbank accession: AAH16450)
11. VACV I4 (Genbank accession: AAO89352)

## Supporting Information

Figure S1 The Δ*F4L* strain has reduced expression of the late VACV protein B5. BSC-40 cells were infected (at a MOI of 5) with wild-type or VACV strains with a deletion of *F4L* (Δ*F4L*) or a Δ*F4L* revertant strain (Δ*F4L* ^REV^). (B) BSC-40 cells were infected as in (A) with wild-type virus or a VACV strain with a deletion of *I4L* (Δ*I4L*). Cells were harvested at the indicated times post-infection and protein extracts were prepared for western blotting. Antibodies against the VACV late protein B5, the early viral proteins F4 and I4 or cellular actin were used for blotting on parallel nitrocellulose membranes. Asterisks indicate mock-infected lysates collected after 24 h.
(9.96 MB TIF)

Figure S2 Expression profile of cellular RR proteins after infection with VACV. HeLa cells were infected with wild-type, Δ*F4L* or Δ*F4L* ^REV^ (revertant) strains (MOI of 5) or were mock-infected (MI). Protein extracts were prepared at the indicated times post-infection and equal amounts of protein were subjected to SDS-PAGE followed by western blotting (WB) for human R1 (HR1), human R2 (HR2), or human p53R2 (Hp53R2). Blots for cellular actin and VACV I3 protein served as loading controls.
(1.03 MB TIF)

Figure S3 Immunoprecipitation of His_6_-tagged F4 with human R1 (HR1). HeLa cells were infected with the indicated strains (MOI of 10) for 8 h and then protein extracts were subjected to immunoprecipitation (IP) with anti-His_6_ antibodies. Western blots (WB) of IP material and total lysates are shown. HC, heavy chain. Note that VACV F4 is ∼37 kDa while Hp53R2 (positive control for HR1 interaction) is ∼43 kDa.
(1.16 MB TIF)

Figure S4 Human and viral RR proteins are localized to the cytoplasm during infection with VACV. (A) Localization of human RR subunits in the absence or presence of infection. HeLa cells were mock-infected (mock) or infected with wild-type VACV (VAC) at an MOI of 5 for 10 h after which coverslips were fixed and stained with antibodies against endogenous human R1 (HR1), R2 (HR2), or p53R2. (B) Localization of recombinant human and VACV RR subunits during infection. HeLa cells were co-infected with the indicated strains (MOI of 5 for each virus) for 10 h after which coverslips were fixed and stained with antibodies recognizing Flag or His_6_ epitopes. Arrows indicate positions of cytoplasmic viral DNA. DIC, differential interference contrast.
(7.92 MB TIF)

Figure S5 Growth properties of selected recombinant strains in BSC-40 cells. Cells were infected at a MOI of 0.03, harvested at the indicated time points, freeze-thawed three times, and tittered on BSC-40 cells. Although the experiments in (A) and (B) were done in parallel, they are separated for clarity purposes and thus the wild-type curve is the same in both graphs. The superscript labels above certain virus strains refer to whether the *I4L* locus was inactivated using pDGloxPKO^INV^ (INV)- or pDGloxPKO^DEL^(DEL)- or pZIPPY-NEO/GUS (pZippy)-based vectors. A superscript “REV” refers to a revertant of the Δ*F4L* strain. All pDGloxPKO-based virsues went through a final, three-round plaque purification procedure in Cre recombinase-expressing U20S cells. Symbols represent mean titers determined in triplicate and error bars represent SD. Some error bars are approximately the same size of the symbols.
(0.31 MB TIF)

Table S1 Differential conservation of *Chordopoxirinae* RR genes.
(0.04 MB DOC)

Table S2 Primers described in Text S1.
(0.04 MB DOC)

Text S1 Supporting Information.
(0.06 MB DOC)
