# Supplementary figures and images for: Vaccinia Virus–Encoded Ribonucleotide Reductase Subunits Are Differentially Required for Replication and Pathogenesis
Source: PLoS Pathog. 2010 Jul 8;6(7):e1000984. doi: 10.1371/journal.ppat.1000984 (PMC2900304; doi:10.1371/journal.ppat.1000984)

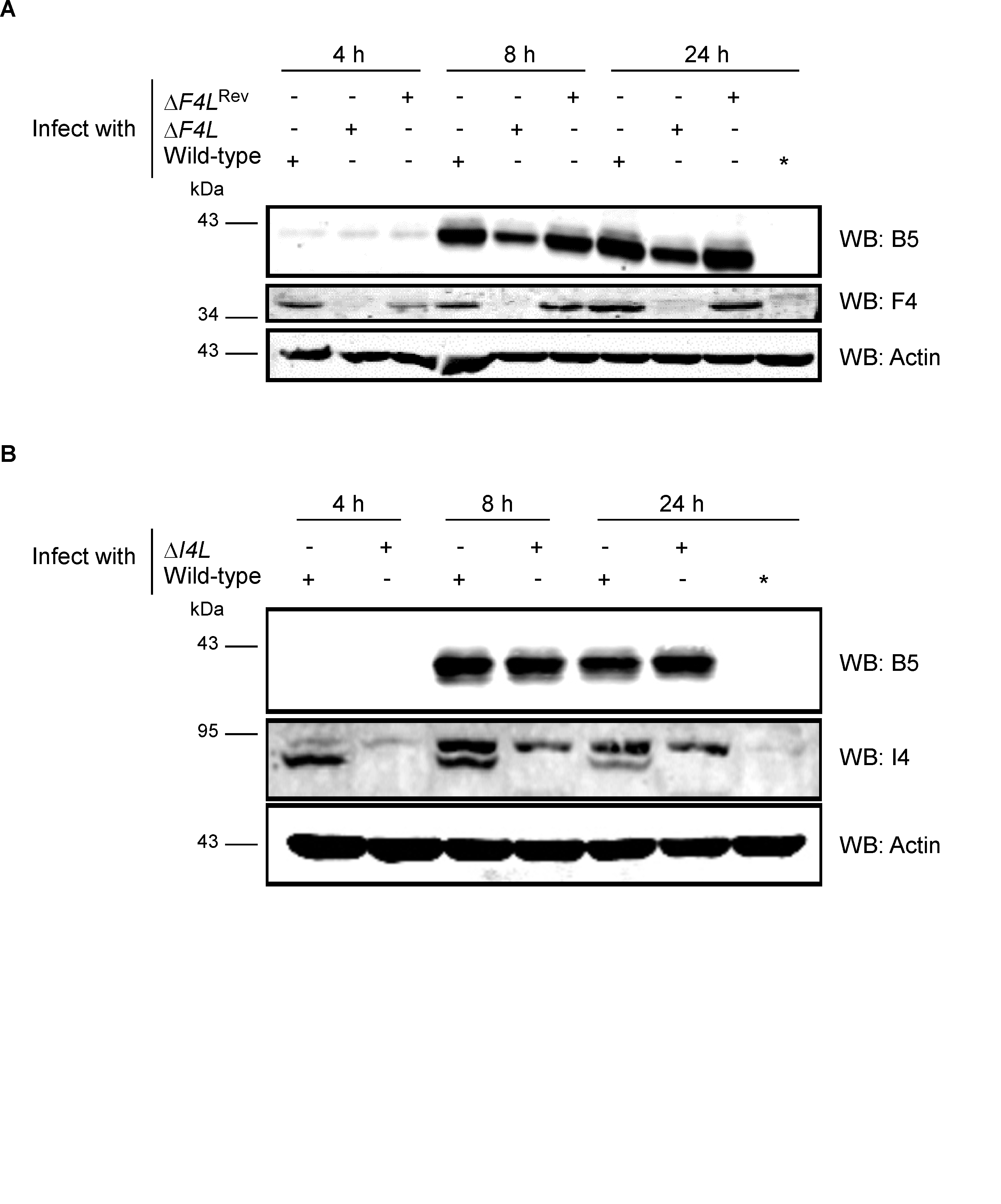

Supplement: Figure S1 — The ΔF4L strain has reduced expression of the late VACV protein B5. BSC-40 cells were infected (at a MOI of 5) with wild-type or VACV strains with a deletion of F4L (ΔF4L) or a ΔF4L revertant strain (ΔF4L REV). (B) BSC-40 cells were infected as in (A) with wild-type virus or a VACV strain with a deletion of I4L (ΔI4L). Cells were harvested at the indicated times post-infection and protein extracts were prepared for western blotting. Antibodies against the VACV late protein B5, the early viral proteins F4 and I4 or cellular actin were used for blotting on parallel nitrocellulose membranes. Asterisks indicate mock-infected lysates collected after 24 h. (9.96 MB TIF) [file ppat.1000984.s001.tif]

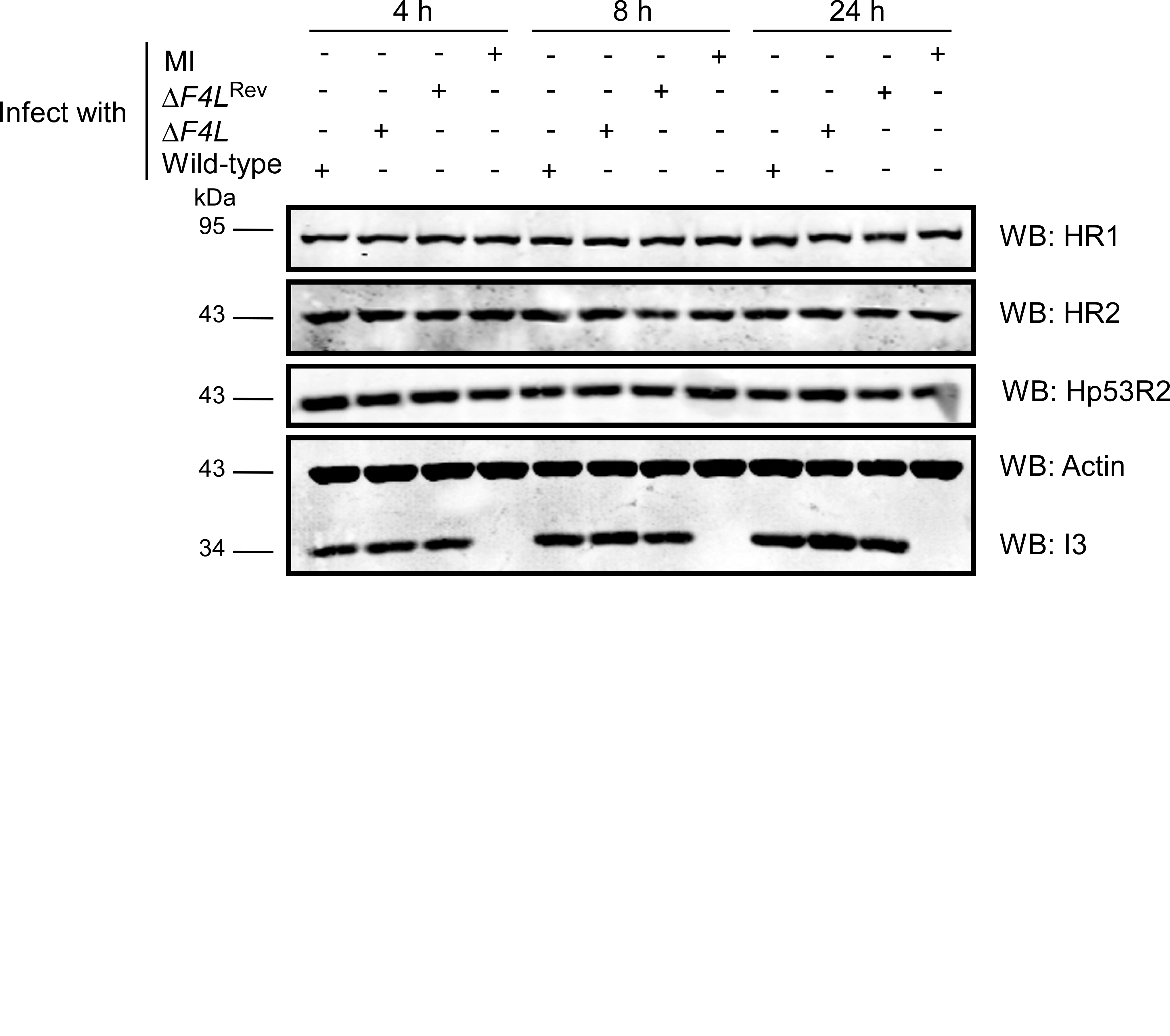

Supplement: Figure S2 — Expression profile of cellular RR proteins after infection with VACV. HeLa cells were infected with wild-type, ΔF4L or ΔF4L REV (revertant) strains (MOI of 5) or were mock-infected (MI). Protein extracts were prepared at the indicated times post-infection and equal amounts of protein were subjected to SDS-PAGE followed by western blotting (WB) for human R1 (HR1), human R2 (HR2), or human p53R2 (Hp53R2). Blots for cellular actin and VACV I3 protein served as loading controls. (1.03 MB TIF) [file ppat.1000984.s002.tif]

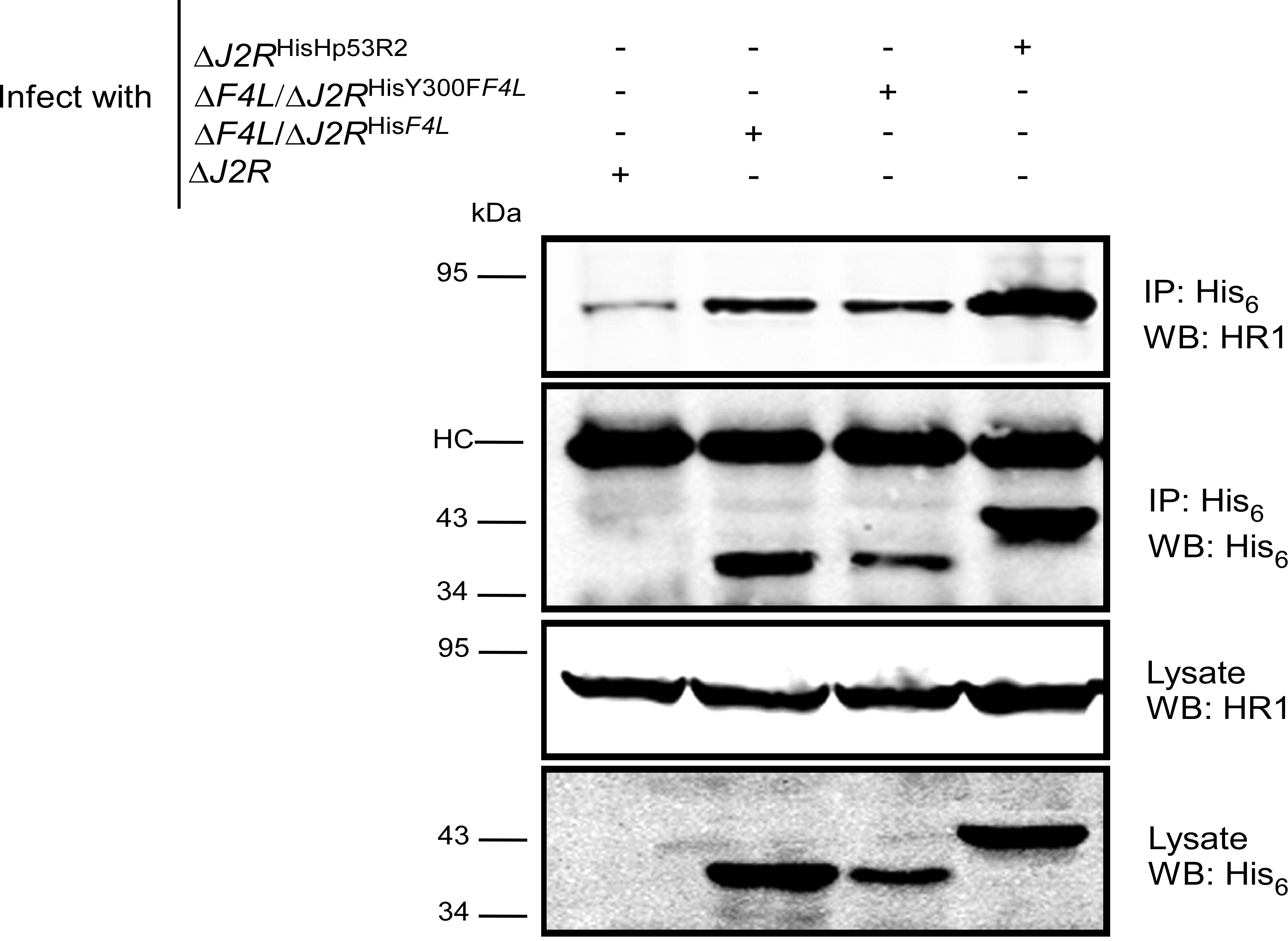

Supplement: Figure S3 — Immunoprecipitation of His6-tagged F4 with human R1 (HR1). HeLa cells were infected with the indicated strains (MOI of 10) for 8 h and then protein extracts were subjected to immunoprecipitation (IP) with anti-His6 antibodies. Western blots (WB) of IP material and total lysates are shown. HC, heavy chain. Note that VACV F4 is ∼37 kDa while Hp53R2 (positive control for HR1 interaction) is ∼43 kDa. (1.16 MB TIF) [file ppat.1000984.s003.tif]

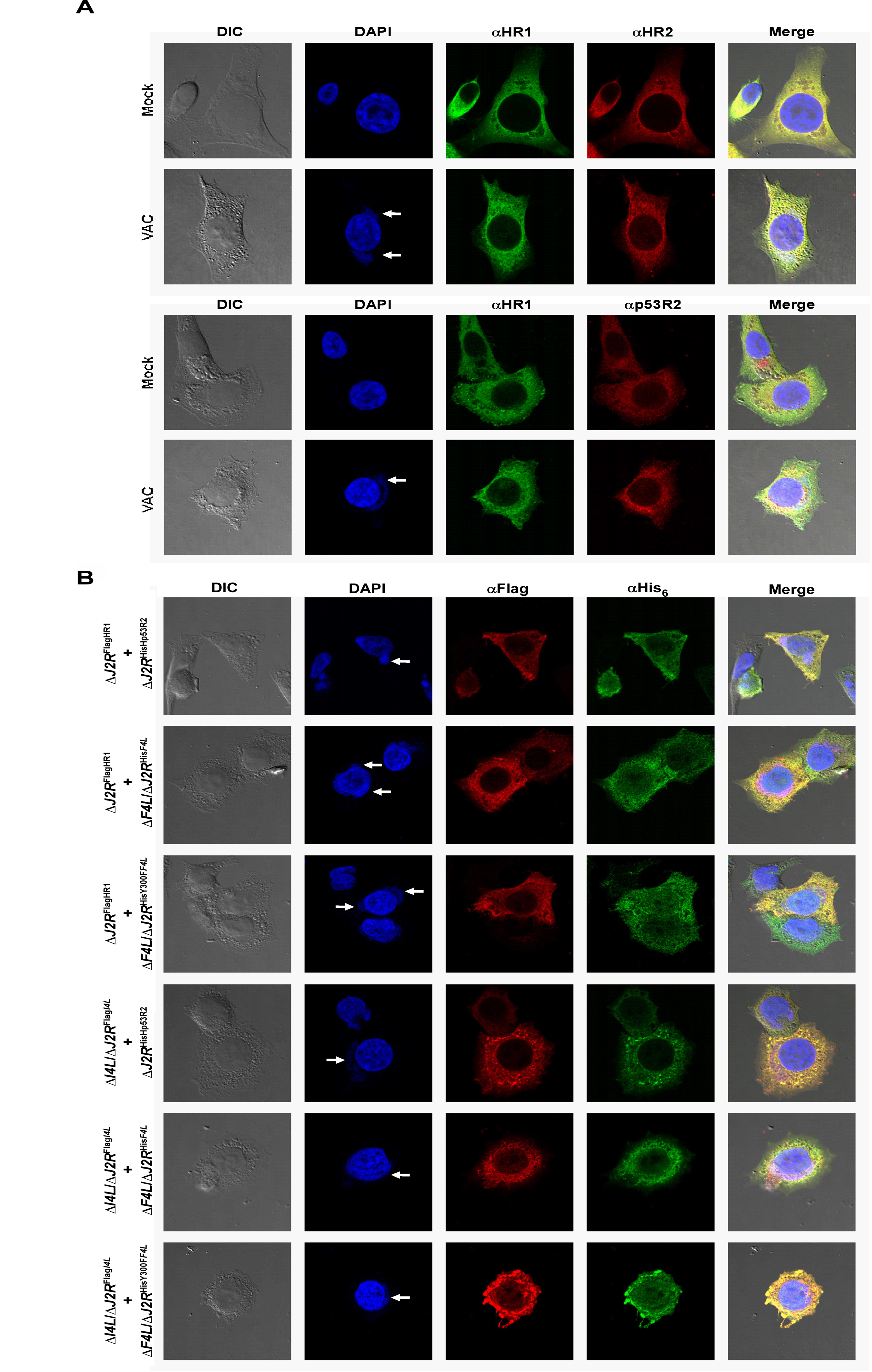

Supplement: Figure S4 — Human and viral RR proteins are localized to the cytoplasm during infection with VACV. (A) Localization of human RR subunits in the absence or presence of infection. HeLa cells were mock-infected (mock) or infected with wild-type VACV (VAC) at an MOI of 5 for 10 h after which coverslips were fixed and stained with antibodies against endogenous human R1 (HR1), R2 (HR2), or p53R2. (B) Localization of recombinant human and VACV RR subunits during infection. HeLa cells were co-infected with the indicated strains (MOI of 5 for each virus) for 10 h after which coverslips were fixed and stained with antibodies recognizing Flag or His6 epitopes. Arrows indicate positions of cytoplasmic viral DNA. DIC, differential interference contrast. (7.92 MB TIF) [file ppat.1000984.s004.tif]

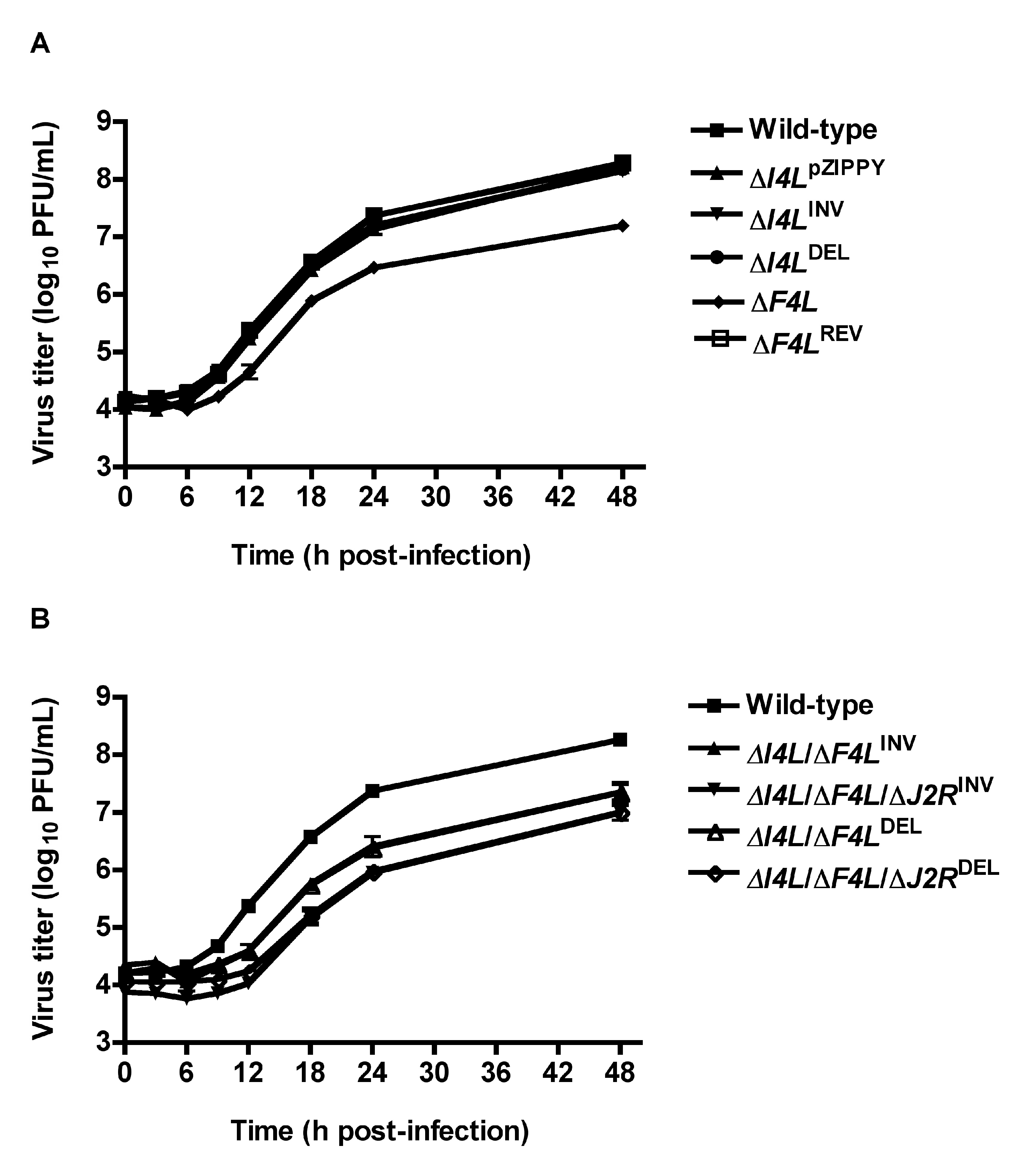

Supplement: Figure S5 — Growth properties of selected recombinant strains in BSC-40 cells. Cells were infected at a MOI of 0.03, harvested at the indicated time points, freeze-thawed three times, and tittered on BSC-40 cells. Although the experiments in (A) and (B) were done in parallel, they are separated for clarity purposes and thus the wild-type curve is the same in both graphs. The superscript labels above certain virus strains refer to whether the I4L locus was inactivated using pDGloxPKOINV (INV)- or pDGloxPKODEL(DEL)- or pZIPPY-NEO/GUS (pZippy)-based vectors. A superscript “REV” refers to a revertant of the ΔF4L strain. All pDGloxPKO-based virsues went through a final, three-round plaque purification procedure in Cre recombinase-expressing U20S cells. Symbols represent mean titers determined in triplicate and error bars represent SD. Some error bars are approximately the same size of the symbols. (0.31 MB TIF) [file ppat.1000984.s005.tif]
