# Supplementary material for: Vaccinia Virus–Encoded Ribonucleotide Reductase Subunits Are Differentially Required for Replication and Pathogenesis
Source: PLoS Pathog. 2010 Jul 8;6(7):e1000984. doi: 10.1371/journal.ppat.1000984 (PMC2900304; doi:10.1371/journal.ppat.1000984)
Supplement: Table S1 — Differential conservation of Chordopoxirinae RR genes. (0.04 MB DOC) [file ppat.1000984.s006.doc]

**Table S1.** Differential conservation of *Chordopoxirinae* RR genes.

| **Genus** | **R1** | **R2** | **TK** | **Example Species** **(A+T%)**^3^ |
| --- | --- | --- | --- | --- |
| *Orthopoxvirus* | +^1^ | + | + | VACV (66.6)  HSPV (66.9)  TATV (66.7)  VARV (67.3) |
| *Suipoxvirus* | + | + | + | SPXV (72.6) |
| *Yatapoxvirus* | - | + | + | TANV (73.1)  YLDV (73.0) |
| *Leporipoxvirus* | - | + | + | MYXV (56.4)  SFV (60.5) |
| *Capripoxvirus* | - | + | + | GTPV (74.7)  SPPV (75.0)  LSDV (74.8) |
| *Cervidpoxvirus* | - | + | + | DPV (73.8) |
| *Avipoxvirus* | - | +^2^ | + | FPV (69.1)  CNPV (69.6) |
| *Molluscipoxvirus* | - | - | - | MCV (35.6) |
| *Parapoxvirus* | - | - | - | ORFV (35.6) |
| Unclassified | - | - | - | CRV (38.8) |

^1^HSPV contains a fragmented R1 gene [80].

^2^FPV contains a fragmented R2 gene [111].

“+” Indicates presence and “-” indicates absence of indicated RR genes in viral genomes.

^3^Example species of indicated genera are given along with average genome A+T content (expressed as a percentage) using Viral Genome Organizer nucleotide content tool for complete genomes [112].

Abbreviations: VACV, vaccinia virus; HSPV, horsepox virus; TATV, taterapox virus; VARV, variola virus; SPXV, swinepox virus; tanapox virus; yaba-like disease virus; MYXV, myxoma virus; SFV, Shope fibroma virus; GTPV, goatpox virus; SPPV, sheeppox virus; LSDV, lumpy skin disease virus; DPV, deerpox virus; FPV, fowlpox virus; CNPV, canarypox virus; MCV, molluscum contagiosum; ORFV, orf virus; CRV, crocodilepox virus.
