# Supplementary material for: Vaccinia Virus–Encoded Ribonucleotide Reductase Subunits Are Differentially Required for Replication and Pathogenesis
Source: PLoS Pathog. 2010 Jul 8;6(7):e1000984. doi: 10.1371/journal.ppat.1000984 (PMC2900304; doi:10.1371/journal.ppat.1000984)
Supplement: Table S2 — Primers described in Text S1. (0.04 MB DOC) [file ppat.1000984.s007.doc]

| **Table S2.** Primers described in Text S1. | |
| --- | --- |
| **Primer No.** | **Sequence (5'-to-3')**^1^ |
| 1 | ACTAGTTAGATAAATGGAAATATCTT |
| 2 | AAGCTTTCAGTTATCTATATGCCTGT |
| 3 | CCGCGGAATCATTTTTCTTTAGATGT |
| 4 | AGATCTTATGATGTCATCTTCCAGTT |
| 5 | ACTAGTTAGATAAATGGAAATATCTT |
| 6 | AGATCTTATGATGTCATCTTCCAGTT |
| 7 | ACTAGTGGAAGGGTATCTATACTTATAGAATAATC |
| 8 | GTCGACTTTTGTTGGTGTAATAAAAAAATTATTTAAC |
| 9 | CCGCGGGGTTAAACAAAAACATTTTTATTCTC |
| 10 | AGATCTGTTTAGTCTCTCCTTCCAAC |
| 11 | AAGCTTATGCATCACCATCACCATCACATGGAACCCATCCTTGCACC |
| 12 | GCGGCCGCTTAAAAGTCAACATCTAAAG |
| 13 | CGAAAAACGTGTGGGTGAATTCCAAAAAATGGGAGTTATGTC |
| 14 | GACATAACTCCCATTTTTTGGAATTCACCCACACGTTTTTCG |
| 15 | GTCGACATGGACTACAAGGACGACGATGACAAG |
| 16 | GCGGCCGCTTAACCACTGCATGATGTACAGATTTCGG |
| 17 | AAGCTTATGGACTACAAGGACGACGATGACAAGATGTTTGTCATTAAACGAAATG |
| 18 | GCGGCCGCTTAACCACTGCATGATGTACAGATTTCGG |
| 19 | GTCGACATGGACTACAAGGACGACGATGACAAG |
| 20 | GCGGCCGCTCAGGATCCACACATCAGACATTC |
| 21 | CCAGTGTGGTGGATGGACTACAAGGACGACGATGACAAGATGCATGTGATCAAGCGAGATG |
| 22 | GCGGCCGCTCAGGATCCACACATCAGACATTC |
| 23 | GGATCCATGCATCACCATCACCATCACATGGGGGACCCGGAAAGGCCG |
| 24 | GCGGCCGCTTAAAAATCTGCATCCAAGG |
| 25 | GAGTTATGTCTCAAGAAGATAATCATTAATCTTTAGATGTTGACTTTTAAG |
| 26 | CTTAAAAGTCAACATCTAAAGATTAATGATTATCTTCTTGAGACATAACTC |
| ^1^Sites where sequences were altered using site-directed mutagenesis are underlined. | |
