# Supplementary material for: Vaccinia Virus–Encoded Ribonucleotide Reductase Subunits Are Differentially Required for Replication and Pathogenesis
Source: PLoS Pathog. 2010 Jul 8;6(7):e1000984. doi: 10.1371/journal.ppat.1000984 (PMC2900304; doi:10.1371/journal.ppat.1000984)
Supplement: Text S1 — Supporting Information. (0.06 MB DOC) [file ppat.1000984.s008.doc]

**TEXT S1**

**Virus construction:**

**∆*F4L* and ∆*F4L*^REV^ strain construction.** The plasmid pZIPPY-NEO/GUS [109] was used to clone an ~500 bp PCR product containing sequences flanking the “*F5L*” side of the *F4L* locus (primers 1 and 2, Table S2) as well as an ~520 bp PCR product containing sequences flanking the “*F3L*” side of the *F4L* locus as well as the last 30 bp of the *F4L* open reading frame (ORF) (primers 3 and 4). The 500 bp PCR fragment was cloned into pZIPPY-NEO/GUS using *Spe*I and *Hin*dIII restriction sites and the 520 bp PCR fragment was cloned into the resulting vector using *Sac*II and *Bgl*II restriction sites. Rescue of this vector (now called pZIPPY-*F5L*^H^+*F3L*^H^) leads to the deletion of nucleotides (nts) 32987-33948 in the WR genome (GenBank accession: NC_006998) comprising 31 nts in the intergenic region between *F5L* and *F4L* ORFs and the first 930 nts of the 960 bp *F4L* ORF. The deleted region is replaced by a p7.5-promoted neomycin resistance (*neo*) gene as well as a bacterial *gusA* gene under the control of a modified H5 promoter. To generate the ∆*F4L* strain, pZIPPY-*F5L*^H^+*F3L*^H^ DNA was transfected into cells infected with wild-type VACV and recombinant viruses were selected by blue coloration in the presence of X-glu.

A ∆*F4L* revertant strain (∆*F4L*^REV^) was constructed by rescue of a cloned PCR product amplified from WR DNA with primers 5 and 6. This PCR product encompasses the same regions of *F5L* and *F3L* used for generating the ∆*F4L* strain as well as the endogenous *F4L* gene. This revertant strain had replication kinetics indistinguishable from wild-type VACV (Figure S5A).

**∆*I4L* and ∆*I4L*/∆*F4L* strain construction.** The plasmid pZIPPY-NEO/GUS was used to clone an ~430 bp PCR product (generated with primers 7 and 8) containing sequences flanking the “*I5L*” side of the *I4L* locus as well as an ~340 bp PCR product (generated with primers 9 and 10) containing sequences flanking the “*I3L*” side of the *I4L* locus. The 430 bp PCR fragment was cloned into pZIPPY-NEO/GUS using *Spe*I and *Sal*I restriction sites and the 340 bp PCR fragment was cloned into the resulting vector using *Sac*II and *Bgl*II restriction sites. These regions of homology were also cloned into a separate vector, pDGloxPKO (see description below) using the same restriction sites. Rescue of the first vector (now called pZIPPY-*I5L*^H^+*I3L*^H^) or the second (now called pDGloxPKO-*I5L*^H^+*I3L*^H^) into VACV leads to the deletion of nts 61929-64240 in the WR genome. The pZIPPY-*I5L*^H^+*I3L*^H^ vector replaces the deleted region with a p7.5-promoted *neo* gene as well as a *gusA* gene under the control of a modified H5 promoter. This vector was used to generate the ∆*I4L* strain. The pDGloxPKO-*I5L*^H^+*I3L*^H^ vector replaces the deleted region with a *yfp*-*gpt* fusion gene promoted by a synthetic early/late poxvirus promoter. Rescue of this vector into the ∆*F4L* background generated the ∆*I4L*/∆*F4L* strain.

The pDGloxPKO vector was synthesized by Geneart (Regensburg, Germany) and is meant to serve as a general knockout vector for poxvirus research. This plasmid contains two multiple cloning sites for insertion of viral DNA homology to flank a gene encoding a fusion protein of yellow fluorescent protein (YFP) and *E.* *coli* xanthine-guanine phosphoribosyltransferase (GPT) protein. This *yfp-gpt* fusion gene is driven by a synthetic early/late poxvirus promoter and permits fluorescence- or MPA-based selection. For the viruses generated here, MPA was used for selection. Two versions of the pDGloxPKO vector exist, one with two identically orientated loxP sites flanking the early/late poxvirus promoter and *yfp-gpt* cassette (pDGloxPKO^DEL^) and an identical vector except one of the loxP sites is inverted relative to the other (pDGloxPKO^INV^). The former vector allows for deletion of the *yfp-gpt* cassette upon passage of the virus in Cre recombinase-expressing U20S cells while the latter leads to the inversion of the *yfp-gpt* cassette and thus does not delete this sequence. For all of the cell culture studies presented in this study, the viruses were generated with the pDGloxPKO^INV^ vector and thus these strains still express the YFP-GPT fusion protein. However, the equivalent strains were produced using pDGloxPKO^DEL^ vectors and it was determined that there was no difference in replication between pDGloxPKO^INV^- and pDGloxPKO^DEL^-based virus strains in culture (Figure S5). A ∆*I4L* strain was also generated using pDGloxPKO^INV^ and pDGloxPKO^DEL^ vectors and it was found that these viruses replicated to titers indistinguishable from the pZIPPY-*I5L*^H^+*I3L*^H^–based recombinant which was used for the experiments presented in this study (Figure S5A). For the mouse pathogenicity studies, the pDGloxPKO^DEL^-based ∆*I4L*/∆*F4L* strain was used which does not express the YFP-GPT cassette (data not shown). Viruses were isolated after transfection of appropriate vectors and selection using either X-glu or MPA in BSC-40 cell culture. All isolates were plaque-purified a minimum of three times in BSC-40 cells and viruses constructed with pDGloxPKO vectors were plaque-purified three more times in Cre recombinase-expressing U20S cells. Deletion of the *I4L* locus and loss of I4 expression was confirmed by PCR (data not shown) and western blotting (Figure 2B).

**Other VACV strains constructed.** All viruses generated in the ∆*I4L*/∆*F4L* background in this and following sections used the strains constructed with the aforementioned pDGloxPKO^INV^ vector. The plasmid pSC66 [108], a derivative of the VACV transfer vector pSC65 [110] was used to insertionally-inactivate the *J2R* locus as well as to introduce foreign genes into the *J2R* locus for expression (see below). This vector contains regions of homology flanking both sides of the *J2R* ORF and creates a disruption in the *J2R* ORF such that an insertion is made in between nts 81001 and 81002 in the WR genome. This ~4 kb insertion encodes a *lacZ* gene under the control of a p7.5 poxvirus promoter as well as introduces a second, early/late synthetic poxvirus promoter that initiates transcription in the opposite direction of the p7.5-*lacZ* cassette [110]. A multiple cloning site downstream of the synthetic promoter allows for the insertion of foreign ORFs to be expressed [110]. Transfection of pSC66 DNA into ∆*I4L*/∆*F4L*, ∆*F4L*, or wild-type VACV-infected BSC-40 cells and subsequent selection of blue plaques (in the presence of X-gal in solid growth media) allowed for the creation of VACV strains ∆*I4L*/∆*F4L*/∆*J2R*, ∆*F4L*/∆*J2R*, and ∆*J2R*, respectively. Disruption of the *J2R* locus was confirmed by PCR analysis (data not shown).

Primers 11 and 12 were used to PCR-amplify and clone a His_6_-tagged *F4L* ORF into pCR2.1 (Invitrogen). A *Kpn*I/*Not*I restriction fragment was then isolated from this plasmid and cloned into the *Kpn*I/*Not*I restriction sites of pSC66 (generating pSC66^His^*^F4L^*). Rescue of pSC66^His^*^F4L^* into the ∆*F4L* background generated strain ∆*F4L*/∆*J2R*^His^*^F4L^* and rescue into the ∆*I4L*/∆*F4L* background generated strain ∆*I4L*/∆*F4L*/∆*J2R*^His^*^F4L^*. Site-directed mutagenesis was performed using primers 13 and 14 and a QuikChange II XL-kit (Stratagene) to generate a His_6_-tagged *F4L* ORF encoding a Y300F substitution (creating pSC66^HisY300F^*^F4L^*). The altered sites in the primers are underlined in Table S2. Rescue of pSC66^HisY300F^*^F4L^* into the ∆*F4L* background generated strain ∆*F4L*/∆*J2R*^HisY300F^*^F4L^* and rescue into the ∆*I4L*/∆*F4L* background generated strain ∆*I4L*/∆*F4L*/∆*J2R*^HisY300F^*^F4L^*.

Primers 15 and 16 were used to PCR-amplify a Flag-tagged *I4L* ORF from a pCR2.1 vector containing a Flag-tagged *I4L* ORF insert previously generated using primers 17 and 18. The resulting PCR fragment was sub-cloned into pCR2.1 and a *Sal*I/*Not*I restriction fragment was cloned into the *Sal*I/*Not*I sites of pSC66 (generating pSC66^Flag^*^I4L^*). Rescue of pSC66^Flag^*^I4L^* into the ∆*I4L* background generated strain ∆*I4L*/∆*J2R*^Flag^*^I4L^*. Primers 19 and 20 were used to PCR-amplify a Flag-tagged HR1 ORF from a pCR2.1 vector containing a Flag-tagged HR1 ORF insert previously generated using primers 21 and 22 and HR1 cDNA (Invitrogen). The resulting PCR fragment was sub-cloned into pCR2.1 and a *Sal*I/*Not*I restriction fragment was cloned into the *Sal*I/*Not*I sites of pSC66 (generating pSC66^FlagHR1^). Rescue of pSC66^FlagHR1^ into the wild-type background generated strain ∆*J2R*^FlagHR1^.

Primers 23 and 24 were used to PCR-amplify a His_6_-tagged Hp53R2 ORF from cDNA (Genecopeia Inc.; Germantown, MD). The resulting PCR fragment was sub-cloned into pCR2.1 and a *Kpn*I/*Not*I restriction fragment was cloned into the *Kpn*I/*Not*I restriction sites of pSC66 (generating pSC66^HisHp53R2^). Rescue of pSC66^HisHp53R2^ into the wild-type background generated strain ∆*J2R*^HisHp53R2^ while rescue into the ∆*F4L* background generated ∆*F4L*/∆*J2R*^HisHp53R2^.

His_6_-tagged R2 genes from the Chordopoxviruses: ECTV strain Moscow (EVM028), MYXV strain Lausanne (m015L), and SFV strain Kasza (s015L) were cloned into pCR2.1 after PCR amplification from viral DNA stocks using appropriate primers. These genes were then subjected to *Sal*I/*Not*I digestion with subsequent cloning into *Sal*I/*Not*I-digested pSC66 generating vectors pSC66^HisECTVR2^, pSC66^HisMYXR2^, and pSC66^HisSFVR2^ and rescue of these vectors into the ∆*F4L* strain produced strains ∆*F4L*/∆*J2R*^HisECTVR2^, ∆*F4L*/∆*J2R*^HisMYXR2^, and ∆*F4L*/∆*J2R*^HisSFVR2^, respectively.

Site-directed mutagenesis was performed using primers 25 and 26 to introduce a premature stop codon (sites changed underlined, Table S2) into the *F4L* genes of pSC66^His^*^F4L^* and pSC66^HisY300F^*^F4L^* vectors. Introduction of this stop codon prevents the expression of the last C-terminal seven residues in VACV F4 which represents the putative R1-binding domain (R1BD) (boxed sequences in Figure 1). Rescue of the resulting vectors, pSC66^His^*^F4L^*^∆R1BD^ and pSC66^HisY300F^*^F4L^*^∆R1BD^ generated strains ∆*F4L*/∆*J2R*^His^*^F4L^*^∆R1BD^ and ∆*F4L*/∆*J2R*^HisY300F^*^F4L^*^∆R1BD^, respectively.
